# FUT9-Driven Programming of Colon Cancer Cells towards a Stem Cell-Like State

**DOI:** 10.3390/cancers12092580

**Published:** 2020-09-10

**Authors:** Athanasios Blanas, Anouk Zaal, Irene van der Haar Àvila, Maxime Kempers, Laura Kruijssen, Mike de Kok, Marko A. Popovic, Joost C. van der Horst, Sandra J. van Vliet

**Affiliations:** Department of Molecular Cell Biology and Immunology, Amsterdam Infection & Immunity Institute, Cancer Center Amsterdam, Vrije Universiteit Amsterdam, Amsterdam UMC, 1081 HZ Amsterdam, The Netherlands; a.blanas@amsterdamumc.nl (A.B.); anouk.zaal@gmail.com (A.Z.); a.i.vanderhaaravila@amsterdamumc.nl (I.v.d.H.À.); maxime.kempers@hotmail.com (M.K.); l.kruijssen@amsterdamumc.nl (L.K.); m.dekok@amsterdamumc.nl (M.d.K.); m.popovic@amsterdamumc.nl (M.A.P.); joost.vanderhorst@gmail.com (J.C.v.d.H.)

**Keywords:** glycosylation, fucosylation, colon cancer, pluripotency, stem cells, drug resistance

## Abstract

**Simple Summary:**

Aberrant glycosylation, for instance heightened expression of fucosylated structures, is a frequent feature observed in tumor cells. Our paper outlines the role of aberrant fucosylation by the Fucosyltransferase 9 (FUT9) as a potent reprogramming factor marking the acquisition of a stem-like state both by murine and human colon cancer cells. Importantly, our study reinforces the implication of aberrant fucosylation in promoting tumor growth and resistance to chemotherapy in the context of colon cancer.

**Abstract:**

Cancer stem cells (CSCs) are located in dedicated niches, where they remain inert to chemotherapeutic drugs and drive metastasis. Although plasticity in the CSC pool is well appreciated, the molecular mechanisms implicated in the regulation of cancer stemness are still elusive. Here, we define a fucosylation-dependent reprogramming of colon cancer cells towards a stem cell-like phenotype and function. De novo transcriptional activation of *Fut9* in the murine colon adenocarcinoma cell line, MC38, followed by RNA seq-based regulon analysis, revealed major gene regulatory networks related to stemness. Lewis^x^, Sox2, ALDH and CD44 expression, tumorsphere formation, resistance to 5-FU treatment and in vivo tumor growth were increased in FUT9-expressing MC38 cells compared to the control cells. Likewise, human CRC cell lines highly expressing FUT9 displayed phenotypic features of CSCs, which were significantly impaired upon *FUT9* knock-out. Finally, in primary CRC FUT9^+^ tumor cells pathways related to cancer stemness were enriched, providing a clinically meaningful annotation of the complicity of FUT9 in stemness regulation and may open new avenues for therapeutic intervention.

## 1. Introduction

Colorectal cancer (CRC) is the fourth most commonly diagnosed cancer type and the third cause of cancer-related deaths in both sexes worldwide [1]. Classification of CRC patients into consensus molecular subtypes (CMS) based on their transcriptional profiles is of great biological and clinical significance, predicting the survival and response to therapy [2]. The current treatment options consist of surgical resection alone (when diagnosed at an early stage) or surgery in combination with systemic administration of cytotoxic chemotherapy for locally advanced and metastatic tumors [3]. So far, the most effective chemotherapy regimen applied to patients with advanced CRC (stages IIB–IV) includes 5-fluorouracil (5-FU), oxaliplatin and irinotecan [4,5]. Moreover, targeted therapy with immune checkpoint inhibitors (e.g., anti-PD1 and anti-CTL4) [6] or monoclonal antibodies (cetuximab and bevacizumab) [7] is often administered in conjunction with chemotherapy and confers a survival advantage to patients with refractory disease [8].

However, the response to therapy remains largely heterogeneous [9] and therapy failure along with eventual relapse and metastatic disease has been attributed to a distinct subpopulation of tumor cells called cancer stem cells (CSCs) [10]. These cells exhibit unique properties, such as self-renewal, infinite division, pluripotency, reduced immunogenicity, resistance to conventional chemotherapy/radiotherapy and tumor-initiating capacity under adverse microenvironmental conditions. Therefore, CSCs are well considered as the relentless engine of tumor evolution [11,12]. Importantly, CSCs are characterized by high plasticity, and their functionality in CRC is predominantly dependent on stimuli derived from the surrounding tumor microenvironment [13]. Although several therapeutic strategies either against CSCs themselves or the so-called CSC niche have recently been proposed, the obstacles that cell plasticity evokes have not been fully overridden yet [14]. Specifically, among the remaining caveats are the lack of CSC-specific phenotypic markers, the limited efficiency of the CSC niche-signal blockade, and the regeneration of the CSC pool upon treatment. Therefore, there is an immense need for the identification of novel molecular pathways defining CSC fate and hardwiring, which can in turn be harnessed for successful therapeutic targeting.

Many of the well-known CSC markers, such as CD44, CD133 (PROM1), LGR5, CD24, EPCAM (ESA), ABCG2, CD34 and CD90/110 are heavily glycosylated [15,16]. Glycosylation is a fundamental post-translational modification of proteins and lipids required for a wide range of biological and cellular functions [17]. Fucosylation is a specific type of glycosylation that is increased in several types of cancer [18]. Overexpression of fucosylated antigens on the surface of cancer cells is linked to increased cell survival and proliferation, epithelial to mesenchymal transition (EMT), metastasis and interaction with endothelial or immune cells [19]. In addition, established cancer biomarkers, such as the carbohydrate antigen 125 (CA-125) [20], carbohydrate antigen 19.9 (CA19.9 or sialyl Lewis^a^) [21] and carcinoembryonic antigen (CEA) [22], are associated with elevated fucosylation levels during cancer progression.

Lewis^x^ (alternatively known as SSEA1 or CD15) is a tumor-associated fucosylated antigen and an established marker of glioblastoma [23] and medulloblastoma [24] stem cells. Among the different fucosyltransferase (FUT) enzymes that can synthesize Lewis^x^, FUT9 is the most competent one regarding Lewis^x^ synthesis both in vitro and in vivo [25] and it is highly conserved between human and mouse [26]. FUT9 is expressed early during embryogenesis [27], dictating the expression of Lewis^x^ in embryonic stem cells (ESCs) [28]. Later in development, the expression of FUT9 is mainly restricted to the stomach and the brain [29], where it is directly implicated in the synthesis of Lewis^x^ and the differentiation of neuronal stem cells [30]. In CRC cells, expression of FUT9 has been associated with the induction of major metabolic changes, and for this reason FUT9 has been proposed as a metabolic driver of advanced-stage CRC [31]. Nevertheless, the impact of FUT9 expression on stemness acquisition by colon cancer cells, along with other functional features of CSCs such as drug resistance, remains ill defined.

Here, we explored the major regulatory programs instructed in colon cancer cells upon FUT9 expression by combining computational and experimental analyses. Our results provide evidence that FUT9 dictates a stem cell-like fate in colon cancer cells both in mice and humans, suggesting a conserved role of this enzyme during malignant transformation and tumor fueling.

## 2. Results

### 2.1. Identification of Gene Regulatory Networks Linking FUT9 Expression to Stemness in Glyco-Engineered Colon Cancer Cells

Since the FUT9 enzyme and its synthesized product, the Lewis^x^ glycan structure, are expressed in embryonic [28] and neuronal [30] stem cells, we hypothesized that their expression might also be associated with stemness in the context of cancer. Preliminary studies have shown an association between FUT9 and CD44 or Oct4 expression in CRC cells [31]. Hence, we set out to assess the role of FUT9 in colon cancer stemness induction. We exploited MC38 as a model system, because it is a well-known murine (C57BL/6 origin) colon adenocarcinoma cell line that is commonly used in preclinical mouse models of CRC [32]. Wild type MC38 (MC38-WT) cells are completely devoid of FUT9 expression, expressing only FUT8, FUT10 and FUT11 (Figure S1A). Additionally, MC38-WT lacks expression of Lewis antigens, such as Lewis^x^ [33]. Therefore, in order to unravel the full range of molecular programming instructed by FUT9 expression in the context of colon cancer, we proceeded with transcriptional induction of the *Fut9* gene in MC38-WT cells using the CRISPR-dCas9-VPR system [33].

**Figure 1:**
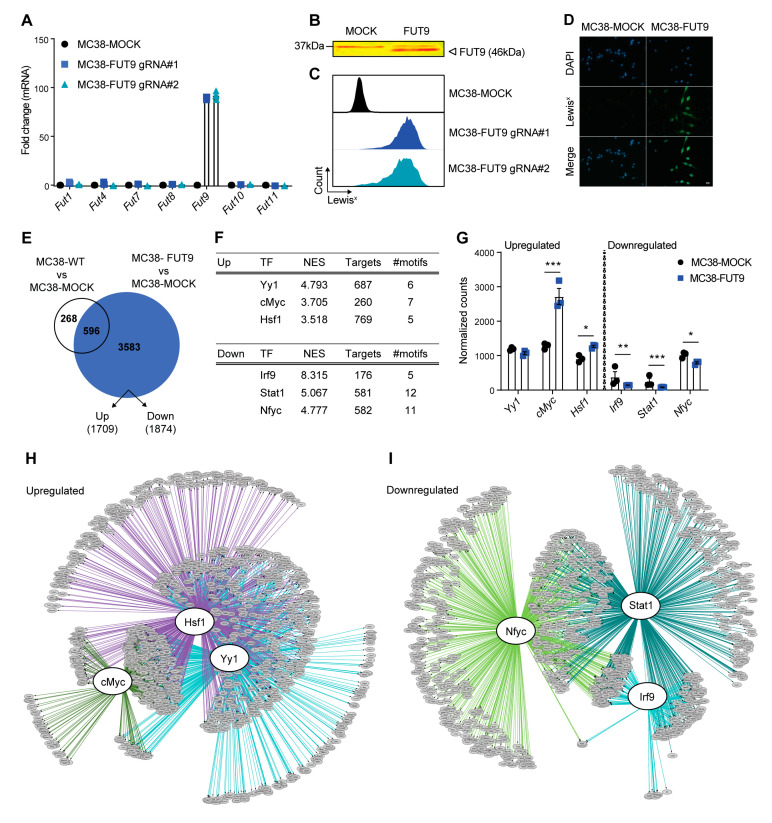
Identification of gene regulatory networks linking FUT9 expression to stemness in glyco-engineered colon cancer cells. (**A**) RT-PCR-based assessment of mRNA levels of murine fucosyltransferases (FUT) upon transcriptional activation of the *Fut9* gene in MC38 cells using the CRISPR-dCas9-VPR technology. Expression was normalized to the housekeeping gene *Gapdh* (*M. musculus*). Differences (fold change) relative to MC38-MOCK cells are depicted in triplicates. Data representative of two independent experiments. (**B**) Western blot analysis for FUT9 in MC38-glycovariants. Data representative of two independent experiments. (**C**) Flow cytometric analysis of the Lewis^x^ expression on the surface of MC38 glyco-engineered cells. Histograms representative of two independent experiments. (**D**) Representative images of anti-Lewis^x^ immunofluorescence staining in MC38-MOCK and MC38-FUT9 cells. DAPI staining represents nucleic acid (nuclear) staining. Scale bar 10 μm. (**E**) Venn diagram depicting the number of differentially expressed genes (DEGs) between MC38-WT vs. MC38-MOCK and MC38-FUT9 vs. MC38-MOCK cells identified by RNA-seq analysis. In MC38-FUT9 cells compared to MC38-MOCK cells, 1709 genes were upregulated and 1874 genes were downregulated. (**F**) The top 3 of the predicted transcription factors (TFs) identified by the RNA-seq-based regulon analysis (iRegulon) corresponding to the upregulated genes (top panel) and the downregulated genes (lower panel) in MC38-FUT9 cells. NES represents the normalized enrichment score. The number of the predicted direct targets among DEGs and the significantly enriched motifs corresponding to each regulator is provided in the table. More details on the motifs and TF-associated genes can be found in Tables S2–S5. (**G**) Normalized counts of the top regulators identified with iRegulon. Statistical significance was assessed using the Benjamini–Hochberg false discovery rate (FDR) <0.05 (* *p* < 0.05, ** *p* < 0.01 and *** *p* < 0.001). (**H**,**I**) Regulatory networks for the upregulated (**H**) and downregulated (**I**) genes found in MC38-FUT9 cells reveal a strong overlap among the regulons and the predicted TFs. Direct targets (DEGs) are in grey circle nodes and TFs in white circle nodes. Regulons for each TF are represented by the different line colors. # indicates the gRNA number.

We generated MC38 cells exhibiting specific and stable expression of FUT9 both at the transcriptional (Figure 1A) and the protein level (Figure 1B) by transfecting the cells with two different guide RNA (gRNA) sequences (gRNA#1 or gRNA#2) targeting the promoter region of the *Fut9* gene. Induction of FUT9 expression resulted in robust neo-expression of the Lewis^x^ antigen on the surface of MC38 cells as assessed by flow cytometry (Figure 1C) and immunofluorescence (Figure 1D), which was absent in the control MC38-MOCK cells. Given that both MC38-FUT9 gRNA#1 and MC38-FUT9 gRNA#2 cells were sorted in bulk to avoid clonal effects and displayed comparable levels of FUT9 induction, we continued with MC38-FUT9 gRNA#1 (hereafter referred to as MC38-FUT9) for the remainder of our studies.

Next, we investigated whether FUT9 expression leads to transcriptional changes in the glyco-engineered MC38 cells, by performing the RNA sequencing (RNA-seq) analysis in MC38-WT, MC38-MOCK and MC38-FUT9 cells (Figure S1C, Table S1). Comparative analysis revealed that the expression of 3583 genes was specifically altered in MC38-FUT9 cells compared to MC38-MOCK cells, yielding upregulation of 1709 genes and downregulation of 1874 genes (Figure 1E, Table S1A,B). These results indicate that FUT9 expression exerts a critical role in shaping the transcriptome of colon cancer cells. As expected, FUT9 expression was one of the most prominent hits in MC38-FUT9 cells, whereas the expression of the other FUTs was unaffected (Figure S1B, Table S1B), which is in line with our RT-PCR expression data (Figure 1A).

We determined the underlying regulatory networks related to the transcriptional changes in MC38-FUT9 cells by exploiting iRegulon, a computational method that detects the master regulons and cofactors from a set of differentially expressed genes (DEGs) using large motif and track collections and subsequently associates the enriched motifs with predicted transcription factor (TF) specificities (Tables S2–S5) [34]. According to this *cis*-regulatory sequence analysis, the top three TFs corresponding to the FUT9-induced upregulated genes were Ying Yang 1 (Yy1), cMyc and Heat shock factor 1 (Hsf1; Figure 1F, top table; Figure S1D; Tables S2 and S3). Yy1 is a well-known TF that governs metabolic programs related both to embryonic stem cell development and cancer initiation [35]. In colon cancer, particularly, Yy1 has a pro-tumorigenic role and it is responsible for high grade metastasis, drug resistance and promotion of stem cell-specific transcriptional programs, including the Wnt/β catenin pathway [36]. Besides Yy1, cMyc [37] and Hsf1 [38] also drive transcriptome rewiring in colon CSCs, eventually leading to tumorigenesis and metastasis. Importantly, the Yy1 gene network is coregulated along with the respective networks of the cMyc and Hsf1 TFs (Figure 1H), highlighting a positive regulation of stemness upon FUT9 induction.

Moreover, as the top-scoring TFs responsible for the downregulated genes upon FUT9 expression in MC38 cells (Tables S4 and S5), we identified the Interferon regulatory factor 9 (Irf9; representative image of the transcription motif in Figure S1E), Signal transducer and activator of transcription 1 (Stat1) and Nuclear transcription factor Y subunit gamma (Nfyc; Figure 1F, bottom table). Despite the limited knowledge that is currently available concerning Nfyc, Irf9 and Stat1 are known to form an interferon (IFN)-response regulon together with Stat2, termed ISGF3 [39]. In addition, decreased IFN/STAT signaling in tumor cells, including the ISGF3 complex, is strongly associated with the acquisition of CSC-like features, resistance to therapy and disease recurrence [40]. Similarly, the extensive overlap among the Irf9, Stat1 and Nfyc regulons underscores their prevalent cofactorship (Figure 1I) and indicates that MC38-FUT9 cells display decreased expression of negative regulators of cancer stemness.

Validation of the expression levels of each of the identified TF revealed that expression of *Myc* and *Hsf1* was significantly increased in MC38-FUT9 cells relative to MC38-MOCK cells, while *Irf9*, *Stat1* and *Nfyc* were significantly downregulated (Figure 1G). The expression of other members of the IFN/STAT pathway that are linked to CRC stemness and aggressiveness was also altered in MC38-FUT9 cells, including downregulation of *Irf1* (Figure S1F) and upregulation of *Stat6* (Figure S1G). Irf1 is negatively regulated by the Wnt/β catenin signaling pathway [41] and suppresses the proliferation and metastatic potential of CRC cells [42]. On the contrary, high Stat6 activity has been associated with the invasive and metastatic capacity of colon cancer cells [43], mainly due to induction of an epithelial to mesenchymal transition (EMT) phenotype.

Taken together, we reverse-engineered the transcriptional regulatory network of MC38-glycovariants via iRegulon and exploited it to elucidate which molecular programs are instructed by FUT9 in colon cancer cells. Specifically, our analysis revealed a coordinated, whole-transcriptome regulatory circuit hinting to stemness reprogramming in MC38-FUT9-expressing cells and warranting further investigation.

### 2.2. FUT9 Neo-Expression Results in an Enhanced Stem Cell-Like Transcriptional Profile and Phenotype

**Figure 2:**
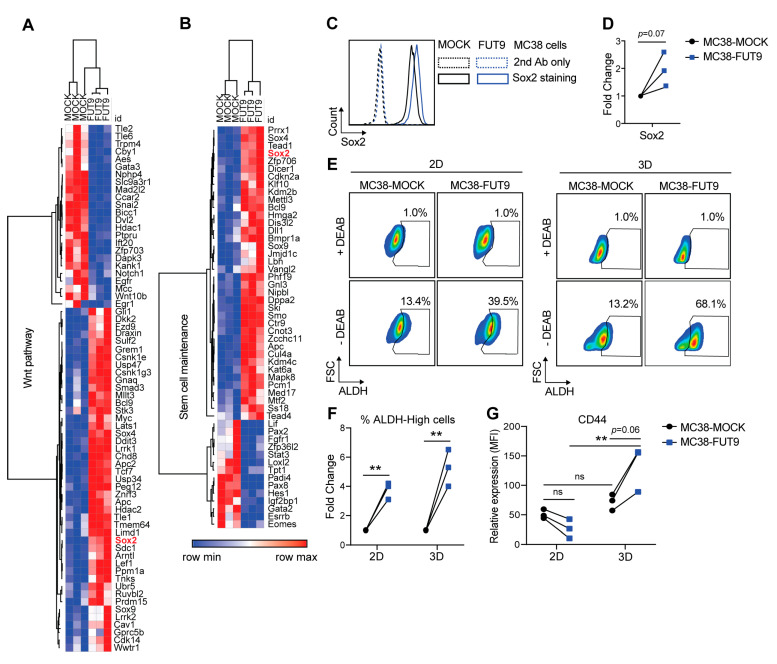
FUT9 neo-expression in MC38 cells results in an enhanced stem cell-like transcriptional profile and phenotype (**A**,**B**) Expression of DEGs associated with the Wnt pathway (**A**) or stem cell maintenance (**B**) in the MC38-MOCK and MC38-FUT9-expressing cells. Sox2 is highlighted in red. (**C**) Intracellular staining of Sox2 in MC38 cells using flow cytometry (left; *n* = 3). (**D**) Fold change in mean fluorescent intensity (MFI) of Sox2 in MC38-FUT9 cells compared to MC38-MOCK cells (*n* = 3). Statistical significance was determined by an unpaired Student’s *t* test. (**E**) ALDH activity in MC38-MOCK and MC38-FUT9 cells cultured either in 2D (left panel) or 3D (right panel), measured using the ALDEFLUOR assay. The DEAB inhibitor of ALDH was used as a control for gating and further analysis of the percentage of ALDH-High cells in each condition. Dotplots are representative of three independent experiments. (**F**) Fold change in the percentage of ALDH-High cells in the MC38-glycovariants upon 2D (*n* = 3) or 3D (*n* = 3) cultures (relative to MC38-MOCK cells). Statistical differences were determined by an unpaired Student’s *t* test (** *p* < 0.01). (**G**) Relative expression (MFI) of CD44 in MC38 cells cultured in 2D (*n* = 3) or 3D (*n* = 3). Statistical significance was determined by a one way ANOVA (ns; no significance, ** *p* < 0.01).

To check for characteristic gene signatures associated with pluripotency and stemness in MC38-FUT9 cells, we used the gene lists provided at the Mouse Genome Informatics (MGI) database (http://www.informatics.jax.org/). Indeed, MC38-FUT9 cells displayed differential expression in genes related to the Wnt pathway (Figure 2A) and stem cell maintenance (Figure 2B), demonstrating that the previously detected regulons were in full agreement with an established stem cell-like transcriptional profile induced upon FUT9 expression. In addition, the Wnt pathway was significantly enriched in our GO term enrichment analysis (Table S6). Furthermore, MC38-FUT9 cells showed differential expression in EMT-related genes (Figure S2A), as well as enrichment for GO terms related to EMT (Table S6).

These results imply that MC38-FUT9 cells might exhibit a potent stem cell-like phenotype in vitro. First, we checked expression of Sox2, Oct4 and Nanog, all implicated in CRC progression and metastasis [10]. Upregulation of Sox2 at the transcriptomic level (Figure 2A,B) also resulted in increased Sox2 protein expression, as determined by flow cytometry (Figure 2C,D). Of note, the nuclear to cytoplasmic (N/C) ratio of Sox2 levels was slightly increased in MC38-FUT9 cells compared to MC38-MOCK cells (Figure S2B). In contrast, protein expression of Oct4 and Nanog, transcriptional cofactors of Sox2 [44], were unaltered upon FUT9 induction in MC38 cells (Figure S2C,D). The combination of Sox2, cMyc and Klf4 was recently reported to be sufficient for reprogramming of mouse somatic cells into induced pluripotent stem cells (iPSCs), even after exclusion of Oct4 from the original Yamanaka cocktail of TFs [45]. Remarkably, besides *Sox2* (Figure 2A,B, Table S1B) and *cMyc* (Figure 1G, Table S1B), *Klf4* was also transcriptionally upregulated in MC38-FUT9 cells compared to MC38-MOCK cells (Figure S2E, Table S1B). Thus, induction of FUT9 neoexpression in MC38 results in upregulation of all the core reprogramming factors involved in stemness.

Another well-known marker characterizing both healthy and CSCs is a heightened ALDH (aldehyde dehydrogenases) activity [46]. In fact, ALDH represents a group of enzymes catalyzing the oxidation of aldehydes to acids, a process that is pivotal for stem cell self-renewal and maintenance [47]. To assess this, we cultured MC38 cells both in conventional monolayers (2D culture) and in tumorspheres/spheroids (3D culture), since the 3D culture has been proposed as an established platform for stem cell modeling [48]. Indeed, the percentage of ALDH-High cells, as assessed using the ALDEFLUOR assay, was significantly higher in MC38-FUT9 cells compared to MC38-MOCK cells after both 2D and 3D culture (Figure 2E lower panels, Figure 2F). Interestingly, the percentage of ALDH-High cells upon 3D culture even reached 68.1% in the MC38-FUT9 cells, while the percentage of ALDH-High cells in MC38-MOCK cells remained around 13% in both culture systems (Figure 2E).

Besides Sox2 and ALDH, expression of several other surface proteins, such as CD44, CD133, Lgr5, CD24, EpCAM and ABCG2, has been associated with stemness features in the context of colon cancer [10]. Nevertheless, from this panel of markers only CD44 is expressed in MC38-WT and MC38-glyco-engineered cells (MuBase database and our RNA-seq analysis, respectively, data not shown [49]). Hence, we evaluated the cell surface expression of CD44 upon FUT9 expression in 2D and 3D. Although CD44 expression was decreased in MC38-FUT9 cells compared to MC38-MOCK cells after 2D culture, MC38 cells cultured in 3D exhibited higher levels of CD44 upon FUT9 expression (Figure 2G). This finding was in line with the ALDH expression profile (Figure 2E) of MC38-FUT9 cells, showing that FUT9 expression boosts the in vitro stem cell-like phenotype of MC38 cells more potently in spheroids than in monolayers.

### 2.3. MC38-FUT9-Expressing Cells Display Functional Properties of Cancer Stem-Like Cells

**Figure 3:**
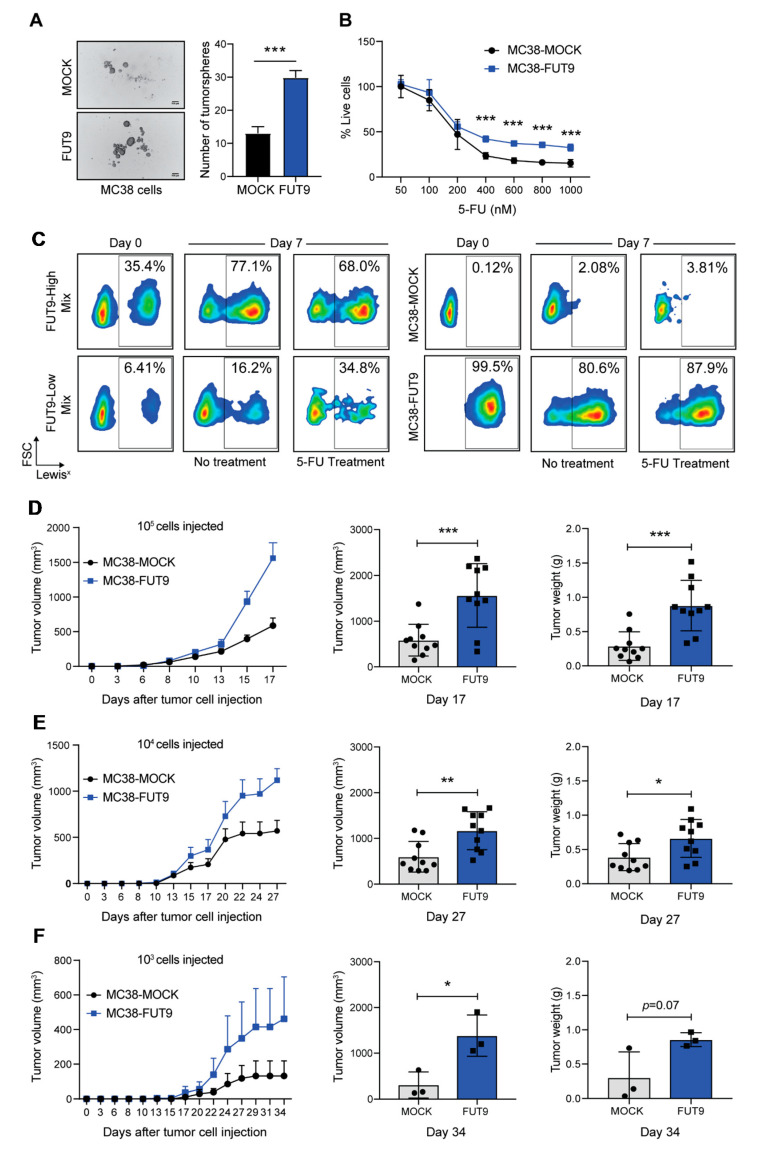
MC38-FUT9 cells display functional properties of cancer stem-like cells. (**A**) Representative images (left) and quantification (right) of tumorspheres formed by MC38 cells during 3D culture. Error bars represent SD. Data are representative of three independent experiments. Statistical significance was determined by an unpaired Student’s *t* test (*** *p* < 0.001). Scale bar 100 μm. (**B**) Viability of MC38-glycovariants upon treatment with indicated concentrations of 5-fluoruracil (5-FU) in 3D culture for 7 days. The percentage of living cells was determined using the CTB fluorometric assay and values were normalized to the corresponding concentration DMSO treatment for each cell line. Error bars represent SD; *n* = 3. Statistical significance was determined by multiple Student’s *t* tests (*** *p* < 0.001). (**C**) Cancer stem cell (CSC)-enrichment assay using flow cytometric analysis of the Lewis^x^ antigen expression on the surface of MC38 cells cultured in 3D either alone (right panel; MC38-MOCK and MC38-FUT9) or in a heterogeneous cell suspension (left panel; FUT9-High Mix and FUT9-Low Mix) in the presence or absence of 5-FU treatment. (**D**–**F**) MC38-MOCK and MC38-FUT9 cells were injected under the skin (left and right flank, respectively) of NSG mice (10 mice per group) at different absolute numbers, 10^5^ (**D**), 10^4^ (**E**) and 10^3^ (**F**), and tumor growth was monitored over time (left panel). The tumor volume during the last measurement (middle panel) and the weight of the isolated tumors (right panel) at day 17, 27 or 34 are depicted. Measurements correspond to the mice that developed both a MC38-MOCK and MC38-FUT9 tumor. Statistical differences were determined by an unpaired Student’s *t* test (* *p* < 0.05, ** *p* < 0.01 and *** *p* < 0.001).

CSCs have the unique ability to grow under adverse environmental conditions [11]. This feature is often recapitulated by the anchorage- and serum-independent growth of CSC-like cells during 3D culture in vitro. Thus, we next examined the tumorsphere forming capacity of our MC38-glycovariants. Interestingly, the number of tumorspheres formed by MC38-FUT9 cells was 3 times higher compared to MOCK cells (Figure 3A), implying that FUT9 expression confers a significant advantage to MC38 cells during anchorage- and serum-independent growth.

To exclude that these differences were caused by intrinsic differences in cell proliferation rates, we analyzed the cell cycle progression (Figure S3A). In spite of the relatively faster cycling of MC38-FUT9 cells at 24 h (lower % of cells in the G0/G1 phase and higher % of cells in the G2/M phase), both MC38-FUT9 and MC38-MOCK finally reached and maintained the same proliferation rate at 48 h (Figure S3A). To validate, we also cultured MC38-glycovariants in 2D or 3D and assessed their proliferation after 3 or 7 days using the CTB fluorometric assay (Figure S3B). Although proliferation rates tended to be lower upon FUT9 expression, no significant differences were observed among the examined cell lines. However, MC38-FUT9 cells outnumbered MC38-MOCK cells after two serial passages in 3D culture, which was indicative of a higher self-renewal capacity (Figure S3C). Collectively, these data demonstrate that MC38-FUT9 cells are superior to MC38-MOCK cells in terms of anchorage- and serum-independent growth, albeit this is not due to a faster proliferation rate.

Inherent resistance to chemotherapy is another well-described trait of CSCs. Hence, we tested whether FUT9 expression protects against 5-FU, the frontline treatment for CRC patients. Strikingly, MC38-FUT9 cells, cultured in 3D, displayed increased resistance to 5-FU treatment across a range of dosages (Figure 3B). So, given the growth advantage and the drug resistance phenotype of MC38-FUT9 when cultured in 3D, we hypothesized that in heterogeneous tumor specimens 5-FU treatment would potentially enrich for low-abundance FUT9^+^ cells bearing a CSC-like phenotype, as stem cells (rare cell population) are assumed to accumulate in patients undergoing chemotherapy. Thus, we prepared heterogeneous suspensions containing a different starting percentage of MC38-MOCK and MC38-FUT9 cells, representing tumors with a high and low number of FUT9-positive cells (FUT9-High and FUT9-Low mix; Figure 3C, left panel). As a control, pure MC38-MOCK and MC38-FUT9 cell suspensions were taken along in the assay (Figure 3C, right panel). Next, cells were cultured in 3D in the presence or absence of 5-FU and the percentage of Lewis^x+^ cells was examined at day 0 and at day 7 using flow cytometry (Figure 3C). Indeed, the percentage of Lewis^x+^ cells doubled in the case of FUT9-High Mix, from 35.4% at day 0 to 77.1% at day 7 in the absence and 68.0% in the presence of 5-FU. Even more impressively, in the case of FUT9-Low Mix (6.41% at day 0) the percentage of Lewis^x+^ cells was 2.5× higher in the absence of 5-FU (16.2%) and 5.4× higher in the presence of 5-FU (34.8%) after 7 days of culture, manifesting that 5-FU treatment contributes to the considerable enrichment of MC38-FUT9 cells in heterogeneous 3D cell suspensions. Moreover, the effect is even more prominent when limited dilution is applied upon 5-FU treatment (Figure 3C, FUT9-Low Mix). This is of utmost clinical significance, since it provides evidence for a chemotherapy-induced expansion of lowly abundant colon CSCs, which eventually lead to disease recurrence and metastasis of CRC patients. Of note, no differences were observed in the control cell cultures, although there was a small decrease in Lewis^x^ expression in the cultures containing MC38-FUT9 cells only.

To explore whether the previously described CSC-like phenotypic and functional properties of MC38-FUT9 cells are translated into enhanced tumor aggressiveness, we examined the in vivo tumor growth using immunocompromised (NSG) mice. MC38-MOCK and MC38-FUT9 cells were injected subcutaneously in the right and left flank, respectively, at different absolute numbers of 10^5^ (Figure 3D), 10^4^ (Figure 3E) or 10^3^ cells (Figure 3F), and tumor growth was monitored over time (Figure 3D–F, left panels). Interestingly, MC38-FUT9 tumors grew significantly faster compared to MC38-MOCK tumors independent of the absolute number of cells injected (Figure 3D–F), indicating that FUT9 expression provides a growth advantage to MC38 cells not only in vitro (Figure 3A,C), but also in vivo.

Conclusively, FUT9 expression in the MC38 colon cancer model led to induction of a stemness-oriented ‘cis-meta-regulon’, a CSC-like transcriptional profile and key phenotypic and functional characteristics of tumor-initiating cells.

### 2.4. FUT9 Expression in Human Colon Cancer Cell Lines is Correlated with High Sox2 Expression and CSC-Like Features

**Figure 4:**
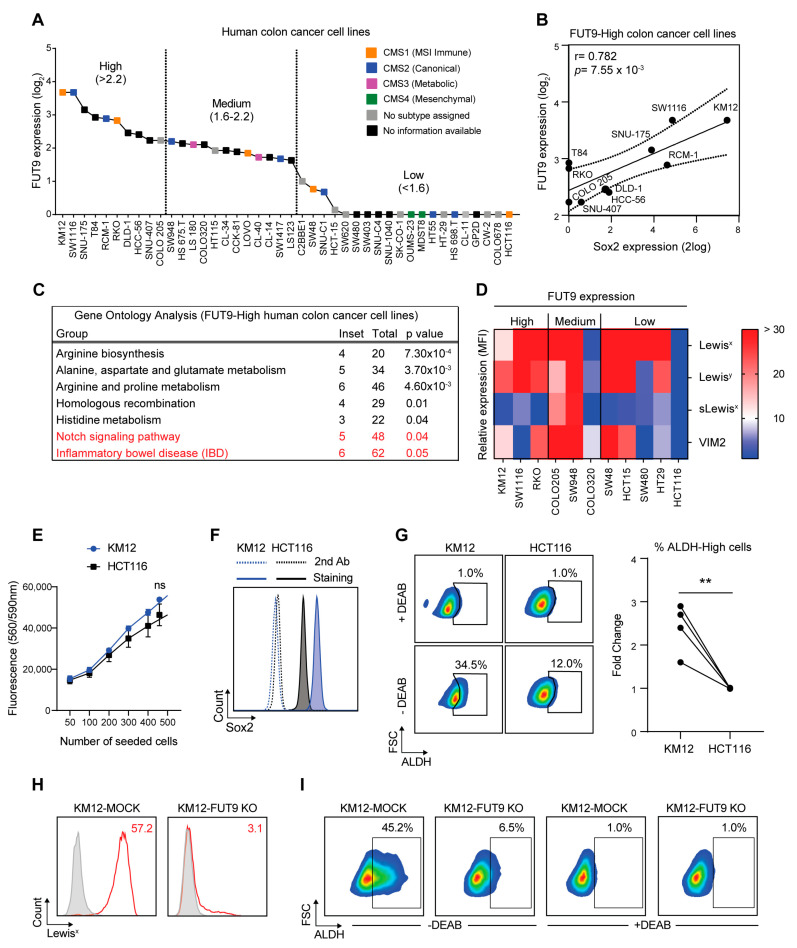
FUT9 expression in human colon cancer cell lines is correlated with high levels of Sox2 expression and ALDH activity. (**A**) Classification of human colon cancer cell lines based on *FUT9* gene expression levels. Data were obtained from the Cancer Cell Line Encyclopedia (CCLE) database. The corresponding molecular subtype for each cell line (if known) is provided [50]. (**B**) Correlation between *FUT9* and *Sox2* gene expression in the subgroup of FUT9-High human colon cancer cell lines. (**C**) Gene Ontology Analysis in FUT9-High human colon cancer cells depicting all the statistically significant (*p* < 0.05) groups. (**D**) Relative surface expression (MFI) of type II Lewis antigens (Lewis^x^, Lewis^y^, sialyl Lewis^x^ and VIM2) on the surface of selected FUT9-High/Medium/Low human colon cancer cell lines, measured by flow cytometry. MFI values were normalized to the binding of the secondary antibody alone. (**E**) Cell proliferation of KM12 and HCT116 cells cultured in 2D at different absolute numbers as determined by the CTB fluorometric assay. Error bars represent SD; *n* = 3. Statistical differences were determined by an unpaired Student’s *t* test (ns; no significance). (**F**) Flow cytometric analysis of intracellular Sox2 expression in KM12 and HCT116 cells cultured in 2D. Dotted lines represent staining with the secondary antibody alone, whereas solid lines represent Sox2 staining. (**G**) Representative dotplots (left) and quantification (right) of ALDH activity in KM12 and HCT116 cells cultured in 2D, measured by the ALDEFLUOR assay. The DEAB inhibitor of ALDH was used as a control for gating and further analysis of the percentage of ALDH-High cells for each condition (*n* = 4). Statistical differences were determined by an unpaired Student’s *t* test (** *p* < 0.01). (**H**) Flow cytometric analysis of Lewis^x^ expression on the surface of KM12 cells glyco-engineered with CRISPR-Cas9. Grey lines represent staining with the second antibody only, red lines represent Lewis^x^ staining. Numbers in red represent the MFI values for Lewis^x^ staining normalized to the binding of the second antibody only. Histograms representative of two independent experiments are shown. (**I**) Representative dotplots of ALDH activity in KM12-MOCK and KM12-FUT9 KO cells measured by the ALDEFLUOR assay. The DEAB inhibitor of ALDH was used as a control for gating and the percentage of ALDH-High cells was calculated for each cell line.

To validate our previous findings obtained from a murine colon cancer model and to translate them into the human setting, we accessed the Cancer Cell Line Encyclopedia database (https://portals.broadinstitute.org/ccle) and categorized human colon cancer cell lines according to their FUT9 expression (Figure 4A). Also, if known, the consensus molecular subtype of each colon cancer cell line was assigned [2,49]. We classified the top 10 cell lines exhibiting FUT9 expression levels >2.2 (2log scale) as ‘FUT9-High’, and made two clusters of ‘FUT9-Medium’ and ‘FUT9-Low’ cell lines with the FUT9 expression levels expression levels ranging from 1.6 to 2.2 and <1.6, respectively. Although there was no clear association between the molecular subtype and FUT9 expression, we did notice that from the ‘FUT9-High’ cell lines, KM12 and RKO belonged to the CMS1 (Immune) subtype, while SW1116 and RCM-1 belonged to the CMS2 (Canonical) subtype. However, these two subtypes were quite prevalent, as they were identified in all three FUT9 clusters. In sharp contrast, the CM3 subtype (metabolic) was detected only in two ‘FUT9-Medium’ cell lines (LS180, CL-40) and the CM4 (mesenchymal) only in two ‘FUT9-Low’ cells (OUMS-23, MDST8).

In analogy to the increased expression of the core-reprogramming TF, Sox2, upon FUT9 expression in MC38 cells (Figure 2C,D and Figure S2A), we wondered whether this pattern was maintained in human cell lines highly expressing FUT9. Indeed, a significant correlation between Sox2 and FUT9 expression could be identified in the ‘FUT9-High’ cluster, with the KM12, SW1116, SNU-175 and RCM-1 cell lines actually expressing the highest levels of both genes (Figure 4B). No significant correlation was found between the expression of Sox2 and FUT9 in the ‘FUT9-Medium’ and ‘FUT9-Low’ clusters (data not shown [51]).

To gain more insight into the pathways associated with high FUT9 expression in human colon cancer cells, we performed a gene ontology analysis in the ‘FUT9-High’ cluster using the R2 genomics analysis and visualization platform [52] (Figure 4C). In this case, the top-scoring hits were predominantly related to metabolism, including arginine, alanine, aspartate, glutamate, proline and histidine biosynthesis. This is in line with previous evidence identifying FUT9 as a metabolic driver of CRC [31]. Among the statistically significant terms, the Notch signaling pathway and inflammatory bowel disease (IBD) were also apparent (highlighted in red). Thus, FUT9 expression in human colon cancer cells is linked both to stemness signaling and an early-driving event (IBD) of this disease.

Based on this, we set out to examine whether the KM12 cell line (which is ‘FUT9-High’ and Sox2-High) displays CSC-specific features in vitro. Notably, KM12 cells express high levels of Lewis^x^ on their surface, along with other FUT9-specific Lewis antigens (such as Lewis^y^ and VIM2), but not sialyl Lewis^x^ (synthesized by the related enzymes FUT3, FUT4, FUT6 and FUT7; Figure 4D). HCT116 cells served as a negative control, because they are subtype-matched (CMS1) to KM12 and contain a defect in the de novo GDP-fucose pathway [53], rendering them unable to synthesize any fucosylated carbohydrate structures, including Lewis antigens (Figure 4D).

Although no apparent differences were observed in the cellular proliferation rates of KM12 and HCT116 cells (Figure 4E), the KM12 cell line expressed higher levels of the Sox2 protein (Figure 4F) and a higher percentage of ALDH-High cells compared to HCT116 cells (Figure 4G). Additionally, relative to HCT116, KM12 cells were more resistant to 5-FU (Figure S4A) across a range of different dosages. To exclude the possibility that this phenotype was 5-FU-specific, we also tested the resistance of KM12 to oxaliplatin and we obtained a similar phenotype (Figure S4B). Taken together, these findings are consistent with the CSC-like phenotype and function of MC38-FUT9 cells, hinting to a positive correlation between FUT9 expression and CSC-like features in human colon cancer cells as well.

To confirm the direct involvement of FUT9 in the CSC-like phenotype of human colon cancer cell lines, we proceeded with CRISPR-Cas9-mediated knock-out (KO) of *FUT9* in the top two FUT9-High cell lines (Figure 4A), KM12 (CMS1 subtype) and SW1116 (CMS2 subtype) cells. In contrast to the KM12 cells, KO of FUT9 in SW1116 cells did not lead to ablation of Lewis^x^ expression on the cell surface (Figure 4H and Figure S4C). On the contrary, we observed an increase in Lewis^x^ expression on SW1116-FUT9 KO cells relative to SW1116-MOCK cells (Figure S4C), possibly due to redundancy among the Lewis^x^-synthesizing FUTs expressed in these cells [54,55]. Additionally, we could detect minor differences in the expression of LGR5 and CD133 between the KM12-MOCK and KM12-FUT9 KO cells, but not between the SW1116 cell lines, while no differences were observed in relation to CD24 expression in the examined cell lines (Figure S4E). Nevertheless, a higher self-renewal potential of KM12-MOCK cells compared to KM12-FUT9 KO cells was noted after two serial passages in 3D culture (Figure S4F–G). Despite the differences between the cell lines mentioned above, both KM12 and SW1116 cells lacking FUT9 expression displayed a remarkable decrease in ALDH activity (Figure 4I and Figure S4D) compared to their MOCK counterparts. Given that ALDH marks pluripotency both in healthy and cancer cells [46], these results suggest indispensable involvement of FUT9 in reprogramming of human colon cancer cell lines towards a stem-like phenotype, a process that seems to be FUT9-specific, but not necessarily Lewis^x^-dependent.

### 2.5. FUT9-Associated Transcriptional Programming of Cancer Stemness in Primary CRC Tumor Cells

**Figure 5:**
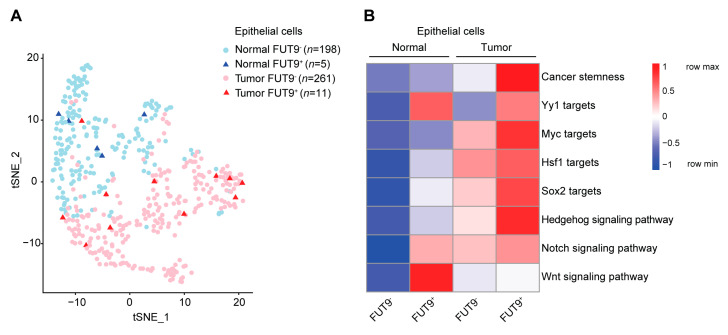
FUT9-specific transcriptional programming of cancer stemness in primary CRC tumor cells. (**A**) Clustering of CRC patient-derived normal and tumor epithelial cells based on FUT9 expression. Expression data were obtained from a previously described single cell-RNA seq analysis [56] and were further analyzed. (**B**) Expression of gene sets related to cancer stemness and pluripotency in the generated FUT9^+^ and FUT9^-^ normal and tumor epithelial clusters.

Finally, to dissect the role of FUT9 in primary CRC, we exploited previously published single-cell transcriptomes derived from 11 CRC primary tumors and their matched normal samples [56]. FUT9 transcripts were almost exclusively found in epithelial cells, and not in fibroblasts, endothelial or immune cells (with the exception of some B cells, data not shown [57]). Therefore, we employed a classification into FUT9^+^ and FUT9^−^ normal or tumor epithelial cell clusters (Figure 5A). Although FUT9^+^ cells did not cluster together, we tested multiple signatures related to cancer stemness [58] and pluripotency (Table S7, Figure 5B). Strikingly, the expression of genes associated with cancer stemness was greatly enhanced in the FUT9^+^ tumor cluster compared to the remaining clusters. In addition, FUT9^+^ cells displayed increased expression of the downstream target genes of Yy1, Myc, Hsf1 and Sox2 [59,60,61], the key TFs linking FUT9 expression to pluripotency in MC38 cells (Figure 1H, Figure 2A–D). This classification further underscores the role of FUT9 in cancer stemness induction. Interestingly, the Yy1 gene regulatory network seemed to be mostly FUT9-specific, but not tumor-specific (Figure 5B). Moreover, the expression of genes involved in the self-renewal and stem cell-maintenance signaling pathways, such as the Hedgehog, Notch and Wnt pathways, followed a differential pattern, whereby the Hedgehog and Notch signaling pathways were upregulated in the FUT9^+^ tumor cluster relative to the FUT9^−^ tumor and the FUT9^+^ normal clusters. In contrast, induction of the Wnt pathway was predominantly found in the FUT9^+^ normal cell cluster, indicating that FUT9 regulates the Wnt signaling pathway in normal human cells. Conclusively, also in primary CRC cells FUT9 expression is correlated with the acquisition of transcriptional programs characteristic of a CSC-like phenotype.

## 3. Discussion

FUT9 is known to dictate stemness in embryonic and neuronal cells [28,30] and it has been proposed to act as a metabolic driver of advanced-stage colon cancer [31]. In the current study, we investigated the regulatory programs instructed in colon cancer cells upon FUT9 expression. Our results provide evidence that FUT9 is responsible for the hardwiring of both murine and human colon cancer cells towards a CSC-like transcriptional profile, phenotype and function, with major implications for tumor growth and resistance to chemotherapy.

Construction of transcriptional regulatory networks using iRegulon [34] has proven its value in the identification of key pathways involved in the regulation of tumor [62] and stromal cells [63] during colon cancer progression. Here, we followed this approach to detect the regulatory circuits of hub genes and TFs that are in play upon transcriptional activation of *Fut9* in the murine colon adenocarcinoma cell line, MC38. Yy1, cMyc and Hsf1 were identified as the master TFs controlling the expression of the upregulated genes in MC38-FUT9 cells (Figure 1H). Moreover, we observed an upregulation of target genes downstream of these TFs in FUT9^+^ human CRC tumor cells (Figure 5B). This intriguing finding is supported by earlier reports showing that Yy1, cMyc and Hsf1 orchestrate early developmental processes that cancer cells hijack during disease progression and metastasis [35,64,65]. Despite the increase in cMyc and Hsf1 expression upon FUT9 expression, no differences in the mRNA levels of Yy1 could be detected among the MC38-glyco-engineered cells (Figure 1G). This might be explained by the fact that Yy1 function is predominantly determined by post-translational modifications, such as *O*-GlcNAcylation [66] and phosphorylation [67].

Many core transcription factors (cMyc, Sox2, Klf4, Oct4 and Nanog), enzymes (ALDH) and key signaling pathways (Wnt, Notch and Hedgehog) are shared between ESCs and CSCs [68]. Our data demonstrate that, besides cMyc, FUT9 expression is correlated with Sox2 expression both in murine (Figure 2A–D and Figure S2B) and human colon cancer cells (Figure 4B and Figure 5B). Sox2 expression in CRC is associated with several CSC features, including spheroid (3D) growth patterns, increased tumor growth and resistance to chemotherapeutic drugs, such as 5-FU [69]. Moreover, Sox2 expression and certain tumorigenic, prosurvival signaling pathways, such as the PI3K/Akt, have been linked before [70]. This is in agreement with all the phenotypic (Figure 2) and functional (Figure 3) CSC-like traits that MC38 cells displayed upon induction of FUT9 expression. Similarly, KM12 cells, highly expressing FUT9 and Sox2 (Figure 4A,B,F), were characterized by enhanced ALDH activity (Figure 4G), as well as increased survival upon treatment with 5-FU (Figure S4A) and oxaliplatin (Figure S4B), relative to HCT116 cells.

Previous studies have implicated the Wnt pathway in the resistance phenotype of human colon cancer cells against 5-FU [71]. Here, we identified differential expression between MC38-MOCK and MC38-FUT9 cells in genes involved in the Wnt signaling pathway (Figure 2A). Furthermore, we observed a vigorous association between FUT9 expression and the Notch (Figure 4C and Figure 5B) and Hedgehog (Figure 5B) signaling pathways in human primary colon cancer cells and cell lines. Activation of these pathways usually coincides with increased ALDH activity [72]. This could possibly explain why de novo expression of FUT9 in MC38 cells resulted in an enhanced frequency of ALDH-High cells (Figure 2E), whereas knock out of FUT9 in KM12 and SW1116 cells diminished the percentage of ALDH-High cells relative to control cells (Figure 4I and Figure S4D). Based on these findings, it becomes evident that FUT9 exerts a central role in shaping molecular programs, which are linked to pluripotency and are commonly found both in ESCs and CSCs.

In general, CSCs are believed to represent a rare cell population that accounts for the 0.01–1% of the bulk tumor [73]. This is in line with the percentage of the FUT9^+^ primary CRC tumor cells (0.04%, 11/272) identified from our scRNA seq reanalysis (Figure 5A). A similar, but 50% lower, percentage (0.02%, 5/203) was detected for the healthy FUT9^+^ cells. Another interesting observation was that FUT9^+^ tumor cells did not cluster together (Figure 5A), indicating that stem-like FUT9^+^ cells display differential gene expression profiles. This phenotype corroborates the heterogeneity and plasticity of CSCs that has been previously described [14]. Additionally, the fact that FUT9^+^ normal cells clustered away from the FUT9^+^ tumor cells highlights the existence of major transcriptional programs differentiating these two cell subsets.

Currently, there is a dire need for identification, functional characterization and validation of biomarkers for cells adopting a CSC-like status. Several fucosyltransferase (FUT) enzymes and their corresponding fucosylated antigens are representative candidates of such biomarkers [19]. For instance, the Lewis^x^ trisaccharide has been proposed as a stem cell selection marker in human glioblastoma cells [74]. Lewis^x^ can be synthesized by different FUTs, albeit FUT9 is the most competent one [25]. In MC38 cells, we have noted induction of a CSC-like state upon neo-expression of both FUT9 and Lewis^x^ (Figure 1A–D). However, the human colon cancer cell lines tested here, KM12 and SW1116, maintained high ALDH activity in a FUT9-specific, but Lewis^x^-independent manner (Figure 4H–I and Figure S4C–D). This finding demonstrates that the fate of each cell type is dictated by the enzyme itself, rather than the synthesized antigen; hence, selected enzymes should be further considered as CSC-specific biomarkers in the future.

Besides FUT9, more glycosyltransferases have been interrelated with stemness induction, including ST6GAL-1, MGAT5 and B4GALT3 [16]. ST6GAL-1 transfers α2-6-linked sialic acids to substrate proteins and its expression is enriched in CD133^+^/ALDH^+^, irinotecan-resistant colon cancer cells [75]. Additionally, in pancreatic and ovarian cancer cells, ST6GAL-1 has been shown to induce the expression of CSC-specific TFs, such as Sox9 and Slug [76]. In colon CSCs, MGAT5 is influencing tumorigenesis and activation of Wnt signaling by synthesizing branched *N*-glycans on the Wnt receptor, FZD-7 [77]. Finally, B4GALT3 regulates stemness through modification of EGFR *N*-glycosylation on the surface of colon CSCs [78].

Aberrant cancer glycosylation drives drug resistance in the context of CRC via different mechanisms, such as alterations in drug absorption, drug metabolism, signaling activation and apoptosis resistance [79]. Here, we show that FUT9 expression is associated with resistance to chemotherapeutic drugs both in murine (Figure 3B) and human (Figure S4A,B) colon cancer cells. Importantly, our results reveal that even a very low (6.4%) starting number of CSC-like cells expressing FUT9 was enough to repopulate the 3D tumor upon treatment with 5-FU (Figure 3C), confirming the strong link between cancer stemness and drug resistance. Besides chemotherapy, CSCs are also resistant to radiotherapy [80] and antibody therapy [81]. So, going forward, the impact of FUT9 expression on resistance acquired by colon cancer cells to other types of therapy remains to be evaluated.

A lot of attention has recently been drawn to cancer immunotherapy and checkpoint inhibition in CRC patients [82]. CSC-specific immunotherapy has been proposed as an alternative and more targeted approach [83]. Colon CSCs have the potential to evade immune surveillance through increased PD-L1 expression [84] and downregulation of MHC-I [85]. In this study, we identified significant downregulation of Irf9 and Stat1, along with their target genes, upon FUT9 expression (Figure 1G,I). Importantly, these TFs are key elements of the ISGF3 complex, which is directly implicated in cancer cell resistance against the antitumor immune response [86]. Therefore, further investigation should focus on the immunomodulatory properties of FUT9-expressing colon cancer cells.

## 4. Materials and Methods

### 4.1. Mice

NOD.Cg-*Prkdc scid Il2rg rm1Wjl*/SzJ female and male mice were purchased from Jackson Laboratory (Bar Harbor, ME, USA). Mice were used at 6–8 weeks of age and maintained in a specific pathogen-free facility at the Amsterdam Animal Research Center (AARC). Mice were randomly assigned to one of the experimental groups. Each group contained 10 mice in total. Experiments were performed in accordance with national guidelines and after approval by the ‘Centrale Commissie Dierproeven’ (CCD) under number AVD1140020173844. Different absolute numbers (10^5^, 10^4^ and 10^3^) of MC38-FUT9 or MC38-MOCK cells were subcutaneously injected in either the right or left flank of each mouse, respectively. Tumor growth was monitored three times per week and total tumor volume was calculated using the formula 4/3 × *π* × *abc* (*a* = width of the tumor/2, *b* = length/2 and *c* = the average of *a* and *b*). Mice were sacrificed when the combined tumor volume from both flanks reached the 1500 mm^3^. Finally, the MC38-MOCK and MC38-FUT9 tumors developed in each mouse were isolated and weighed.

### 4.2. Cell Culture

Wild type MC38 cells (murine colon adenocarcinoma; gift from Prof. Dr. M. van Egmond, Amsterdam UMC, Vrije Universiteit Amsterdam, Department of Surgery and Molecular Cell Biology and Immunology, Cancer Center Amsterdam), wild type HCT116 cells (human colon adenocarcinoma; gift from Prof. Dr. Remond Fijneman, Netherlands Cancer Institute, Department of Pathology) and the respective glyco-engineered cell lines were cultured in monolayers (2D culture) and maintained in DMEM (Gibco, Waltham, MA, USA, 41966-029) supplemented with 10% FBS premium (Biowest, Nuaillé, France, S182B) and 1% penicillin/streptomycin (Gibco, 15140-122). Wild type KM12 (human colon adenocarcinoma; gift from Prof. Dr. Remond Fijneman, Netherlands Cancer Institute, Department of Pathology), SW1116 cells (human colorectal adenocarcinoma; gift from Mrs. Ing. G.W van Pelt, Leiden UMC, Department of Surgery) and the corresponding glyco-engineered cell lines were cultured in monolayers (2D culture) and maintained in RPMI 1640 Medium (Gibco, 31870-025) supplemented with 10% FBS premium (Biowest, S182B), 1% L-glutamine (Gibco, 25030-024) and 1% penicillin/streptomycin (Gibco, 15140-122). Cells were detached using Trypsin-EDTA (Gibco, 15400-054). Cells were maintained at 37 °C and 5% CO_2_ in a humidified incubator, tested for mycoplasma infection monthly. For anchorage-independent growth and tumorsphere formation (3D culture), MC38 and KM12 cells were seeded in 96- or 24-well Corning Costar clear flat bottom ultra-low attachment plates (Merck, Kenilworth, NJ, USA, CLS7007 and CLS3473) at a concentration of 500 or 2000 cells per well, respectively, and cultured in serum-free DMEM-F12 (Thermo Fisher, Waltham, MA, USA, 11,320,033) supplemented with basic Fibroblast Growth Factor (FGF) (20 ng/mL; Thermo Fisher, 13,256,029) and 1× B27 (Thermo Fisher, 17,504,044). Medium was replaced at day 4 to replenish nutrients. At day 7, tumorspheres were dissociated with an enzyme-free cell dissociation buffer (Gibco, 13,151,014), passed through a 70 µm cell strainer (Corning, NY, USA, 352,350) and single cell suspensions were used for further experiments. During serial passages, tumorspheres were dissociated using a tumor dissociation kit (Miltenyi Biotech, Bergisch Gladbach, Germany, 130-095-929), passed through a 70 µm cell strainer (Falcon, 352,350) and the number of single cells was determined after manual counting. Cells were maintained at 37 °C and 5% CO_2_ in a humidified incubator.

### 4.3. Generation of Glyco-Engineered Colon Cancer Cell Lines

The CRISPR/dCas9-VPR constructs used for transcriptional activation of the murine *Fut9* gene were designed and made as previously described [33]. The gRNA sequences targeting the murine *Fut9* promoter region were: FUT9 gRNA#1 GCATATCGGAGACGCAGCAA and FUT9 gRNA#2 GCCTCCCGACTCAACACACG. Similarly, the CRISPR/Cas9 construct used for knocking-out the human *FUT9* gene was made as previously described [87,88]. The following gRNA sequence targeting the human *FUT9* gene was designed and used: FUT9 KO: GCATTGAAATCCATACCTAC. MC38 cells were transfected and selected with 6 μg/mL Puromycin (Invivogen, ant-pr-1) as previously reported [33]. Human CRC cells were transfected in 6-well plates seeded with 100,000 cells per well. Of the empty pSpCas9(BB)-2A-Puro plasmid 2.5 μg (Addgene #62,988; MOCK cells) or the plasmid containing the cloned gRNA sequence (FUT9 KO cells) were delivered to the corresponding wells together with lipofectamine LTX (Thermo Fisher, 15338-100), according to the manufacturer’s instructions. Stable human cell lines were selected with 1 µg/mL puromycin (Invivogen, ant-pr-1) and selection was applied to the cells 48 h post-transfection. Manual cell separation (MACS) with the use of anti-CD15 (Lewis^x^) microbeads (Miltenyi Biotec, 130-046-601) and LS-Columns (Miltenyi Biotec, 130-042-401) was performed according to the manufacturer’s instructions to enrich for the Lewis^x+^ or Lewis^x−^ cell fraction of the glyco-engineered murine and human colon cancer cell lines. All cell lines were selected in bulk in order to exclude potential clonal effects and to mimic tumor heterogeneity.

### 4.4. qRT-PCR Analysis

MC38 cell lysis and mRNA isolation was performed with the mRNA capture kit (Roche, Basel, Switzerland, 11,787,896,001) and cDNA synthesis was performed using the Reverse Transcription System Kit (Promega, Madison, WI, USA, A3500), according to the manufacturer’s instructions. Synthesized cDNA samples together with the KAPA SYBR^®^ FAST qPCR Master Mix (2×) Universal (KAPA BIOSYSTEMS, KK4618) and specific qPCR primers were utilized for each qRT-PCR reaction. Precisely, the qRT-PCR primer sequences used for cDNA amplification were the following ones: *Gapdh* forward primer: CCTGCACCACCAACTGCTTAG; *Gapdh* reverse primer: CATGGACTGTGGTCATGAGCC; *Fut1* forward primer: CGACACAAAGACCCCATCTT; *Fut1* reverse primer: GAAGCCAAAGGTGCCAATAG; *Fut4* forward primer: CAGCCTGCGCTTCAACATC; *Fut4* reverse primer: CGCCTTATCCGTGCGTTCT; *Fut7* forward primer: CCATCCTTATCTGGCACTGG; *Fut7* reverse primer: GCTCCGGTTAGCACTCAGAC; *Fut8* forward primer: GCCAAAATGCCCACAATC; *Fut8* reverse primer: GTTTCCAGCCACACCAATG; *Fut9* forward primer: ATCCAAGTGCCTTATGGCTTCT; *Fut9* reverse primer: TGCTCAGGGTTCCAGTTACTCA; *Fut10* forward primer: GGAGGGAGAGCCTAAACACC; *Fut10* reverse primer: CTACCAGCATCCACCTTTGTC; *Fut11* forward primer: GTCGTCGCACATGAACTGTC and *Fut11* reverse primer: GCTTCCCCCTGATAGAGACC. qRT-PCR for individual genes was ran and analyzed on the CFX96 Real-Time PCR Detection System (BIORAD, Hercules, CA, USA), with all target gene expression levels normalized to *Gapdh* (*M. Musculus*).

### 4.5. Immunoblotting

Cell lysates were prepared using the RIPA buffer (Thermo Fisher, 89,900) supplemented with protease (Thermo Fisher, 78,430) and phosphatase inhibitors (Thermo Fisher, 78,420). Protein concentrations were determined following the instructions of the Pierce BCA Protein Assay kit (Pierce, 23,225). Lysates were boiled with 2× Laemmli buffer (Bio-Rad, Hercules, CA, USA, 1,610,737) for 10 min at 95 °C. Precision Plus Protein Dual Color Standards (Bio-Rad, 1,610,374) were used as the protein ladder. Equal amounts of proteins were subjected to SDS-PAGE on 15% gels and then transferred to PVDF membranes (Bio-Rad, 162-0177). Membranes were blocked with the Odyssey Blocking Buffer in PBS (LI-COR, Lincoln, NE, USA, 927-40000) and then incubated with anti-FUT9 (1:1000; Proteintech, Rosemont, IL, USA, 60230-1-Ig) and anti-beta actin (1:10,000; Proteintech, 66009-1-Ig) in the Odyssey Blocking Buffer containing 0.1% Tween 20 for 1.5 h at room temperature. Membranes were washed 4× with PBS containing 0.05% Tween 20. For fluorescence detection, the following secondary antibodies were used: goat anti-rabbit IgG, IRDye 680 conjugated (1:15,000; LI-COR, 926-68071) for the detection of the anti-beta actin primary antibody and goat anti-mouse IgG, IRDye 800 conjugated (1:15,000; LI-COR, 926-32210) for the detection of the anti-FUT9 primary antibody. Membranes were incubated with secondary antibodies in the Odyssey Blocking Buffer containing 0.1% Tween 20 and 0.01% SDS for 1 h at room temperature. The image was recorded by an Odyssey Imaging System (LI-COR) and further analysis in the pseudo color mode was performed using the Image Studio Lite software (LI-COR).

### 4.6. Flow Cytometry

Cells were harvested, washed and resuspended at a concentration of 1 × 10^6^ cells/mL in phosphate buffered saline (PBS) containing 0.5% bovine serum albumin (BSA; Fitzgerald Industries, Acton, MA, USA, 30-AB75) and 0.02% sodium azide. For extracellular staining, 50,000 cells (50 μL) were added to each well of a 96-well V-bottom plate (Merck, M9686). For intracellular staining, 100,000 cells (100 μL) were added to each well of a 96-well V-bottom plate (Merck, M9686), fixed/permeabilized in 100% methanol for 20 min at −20 °C and washed prior to staining. Cell suspensions were stained for 30 min on ice with anti-Lewis^x^ (1:40, Merck, Kenilworth, NJ, USA, 434,631), anti-Sox2 (1:100; GeneTex, Irvine, CA, USA, GTX101507), anti-Oct4 (1:200; GeneTex, GTX100622), anti-Nanog (1:200; GeneTex, GTX627421), anti-Lewis^y^ (1:20, GeneTex, GTX23359), anti-sialyl Lewis^x^ (1:100, BD Pharmingen, Franklin Lakes, NJ, USA, 551344), anti-VIM2 (1:40, LabNed, Amstelveen, the Netherlands, LN1302156), isotype-APC (1:200; ImmunoTools, Friesoythe, Germany), anti-CD44-APC (1:200; ImmunoTools, 21,850,446 × 2), anti-LGR5-PE (1:100; Biolegend, 373,803), anti-CD133-BV421 (1:50; Biolegend, 372,808) and anti-CD24-AF647 (1:50; Biolegend, 311,110). 7-Aminoactinomycin D (7-AAD; 1:1000; Molecular Probes, A1310) was used for live/dead cell exclusion. Cells were washed and resuspended in 50 μL PBA (PBS, 0.5% BSA and 0.02% sodium azide) containing goat anti-mouse IgM-FITC (1:50; Jackson ImmunoResearch, Cambridge, UK, 115-096-020) for detection of the anti-Lewis^x^, anti-Lewis^y^, anti-sialyl Lewis^x^ and anti-VIM2 primary antibodies. Cells were washed and resuspended in 50 μL PBA containing donkey anti-rabbit IgG (H+L)-AF647 (1:400, Thermo Fisher, A-31573) for detection of the anti-Sox2 and anti-Oct4 primary antibodies or goat anti-mouse IgG (H+L)-AF647 (1:400, Thermo Fisher, A-21236) for detection of the anti-Nanog primary antibody. Cell suspensions were stained for 30 min on ice with the corresponding secondary antibodies. Cells were washed with 100 μL PBA and subsequently resuspended in 100 μL PBA. Fluorescence intensities were measured using a Cyan ADP (Beckman Coulter Brea, CA, USA) or LSRII (BD Biosciences, Franklin Lakes, NJ, USA) flow cytometers. Cytometric data analysis was performed with the FlowJo V10 software (Tree Star, Ashland, OR, USA).

### 4.7. Immunofluorescence

3 × 10^4^ MC38 cells were seeded in each well (200 µL medium/well) of an 8-well chamber slide (µ-Slide 8 well, IBIDI, 80,826) and cultured overnight at 37 °C and 5% CO_2_ in a humidified incubator. The next day the medium was aspirated and cells were washed 3 times with PBS. Cells were fixed with 2% paraformaldehyde (PFA) in PBS for 20 min at RT and washed 3 times with PBA prior to staining. Cells were stained for 1 h at 37 °C with anti-Lewis^x^ (1:80, Calbiochem, 434,631) or anti-Sox2 (1:100; GeneTex, GTX101507) and then washed 3 times with PBA. Next, cells were stained for 1 h at 37 °C with goat anti-mouse IgM-FITC (1:50; Jackson ImmunoResearch, 115-096-020) for detection of the anti-Lewis^x^ antibody and donkey anti-rabbit IgG (H+L)-AF647 (1:400, Thermo Fisher, A-31573) for detection of the anti-Sox2 antibody and then washed 3 times with PBA. Cells were incubated with DAPI (1:3000, Thermo Fisher, D1306) for 20 min at RT and washed 3× with PBA. Finally, cells were mounted with MOWIOL 4-88 (Calbiochem, 475904). Representative images of the Lewis^x^ staining were obtained using the Axio Imager D2 (Zeiss, Germany) microscope (40× objective). For the intracellular Sox2 staining, pictures were taken with the Nikon Ti2 microscope using the 40× objective. Different Z-stack values with steps of 0.4 µm ranging from −0.4 to +1.6 µm were used for deconvolution. Images were taken from 15 different fields of view for each condition. The mean fluorescence intensity was calculated with the NIS-Elements software after deconvolution. The nuclei of the cells were delineated using the DAPI staining (blue channel) and the mean fluorescence intensity was calculated by measuring the red signal (Sox2 staining). The cytoplasm fluorescence intensity was calculated by subtracting the whole red signal from the nuclei red signal. The average and the standard deviation of the fields of view were calculated.

### 4.8. mRNA Library Preparation, RNA-Sequencing, Alignment and Differential Expression Analysis

The mRNA library was prepared as described previously from three independent passages (passages 6–9) of the MC38-MOCK and MC38-FUT9 harvested at three independent time points [89,90]. RNA extraction, library synthesis, as well as the RNA sequencing were performed as previously described [90]. Reads were aligned to the Ensemble *M. musculus* genome (build GRCm38.90) using HiSat2 (v2.0.4) (http://daehwankimlab.github.io/hisat2/) and subsequent processing was performed with samtools (v0.1.19) (http://www.htslib.org/). FeatureCounts (R package Subread v1.5.0-p3, http://subread.sourceforge.net/) was used to quantify aligned reads, excluding multioverlapping reads.

Library size adjustment, trimmed mean of M-values (TMM) normalization and differential expression analysis was done using the R package edgeR (v3.18.1) (https://www.bioconductor.org/packages//2.7/bioc/html/edgeR.html) software. Multidimensional scaling (MDS) plots were used to visualize sample distribution among MC38 cell lines. For differential expression analysis, the negative binomial dispersion was shrunken towards the common dispersion. EdgeR’s exact test for two-group comparison was used for computing *p*-values. Statistical differences in mRNA expression were identified using the following pairwise comparisons: (1) MC38-WT vs. MC38-MOCK cells and (2) MC38-FUT9 vs. MC38 MOCK cells. Here, per comparison, genes with more than 4 zeros across the 6 samples were discarded a priori. Significance was assessed using Benjamini–Hochberg false discovery rate (FDR) <0.05. Sequencing data will be made publicly available at the Sequence Read Archive (SRA) Gene Expression Omnibus thought GSO Series accession number GSE143700 upon acceptance of the manuscript.

### 4.9. Comparative Analysis of Gene Sets

Comparative analysis was performed among the differentially expressed genes (DEGs) in the (1) MC38-WT vs. MC38-MOCK cells (864 genes, Table S1A) and (2) MC38-FUT9 vs. MC38 MOCK cells (4118 genes; Figure S1C) using Venny (v2.1.0, https://bioinfogp.cnb.csic.es/tools/venny/index.html). Of the 4118 DEGs in the MC38-FUT9 cell line, 3522 genes were specifically affected in MC38-FUT9. There were 596 genes that overlapped between comparison of (1) MC38-WT vs. MC38-MOCK and comparison of (2) MC38-FUT9 vs. MC38-MOCK. Analysis of the direction of log_2_ fold change of these 596 overlapping DEGs, however, revealed that 61 of these DEGs showed a different direction of change between the two datasets, meaning that although these genes were differentially expressed in both datasets, differential gene expression in the FUT9 cell line was not the result of glyco-engineering and subsequent differences in cell culturing, as this would otherwise have led to a similar direction of change in gene expression. Therefore, analysis of the 596 overlapping genes yielded an additional 61 DEGs that were included for further analysis. Together, this resulted in 3583 DEGs in the MC38-FUT9 cells for further analysis, of which the expression of 1874 genes was suppressed and the expression of 1709 genes was increased in MC38-FUT9 cells compared to MC38-MOCK cells (Figure 1E, Table S1B).

### 4.10. Motif Enrichment Analysis

To determine whether the 3583 DEGs in the MC38-FUT9 cell line are subject to a gene regulatory network, motif enrichment analysis and transcription factor prediction analysis was performed with Cytoscape v3.6.0 (https://cytoscape.org/), and the iRegulon plugin v1.3 [34], using default settings. Analysis was performed separately for the upregulated and suppressed DEGs.

### 4.11. Gene Ontology Term Enrichment and Gene Signature Analysis

The gene ontology (GO) term enrichment analysis was performed on the 3583 DEGs by MC38-FUT9 cells using Cytoscape v3.6.0 (https://cytoscape.org/) and the ClueGO plugin v2.5.0 (http://apps.cytoscape.org/apps/cluego). Significantly enriched GO terms (Benjamini–Hochberg correction and false discovery rate (FDR) <0.05) were determined with the ontology source GO_BiologicalProcess-EBI-UniProt-GOA, and were subsequently visualized using view style groups, GO level 6–13 and a kappa score threshold of 0.4. For the gene signature analysis, GO term-associated genes were extracted from the Mouse Genome Informatics (MGI) database and compared to our gene set. Gene lists for the following GO terms were used; canonical Wnt signaling pathway, stem cell population maintenance and epithelial to mesenchymal transition. Normalized counts from our RNA-seq analysis of GO term-associated genes were visualized in heatmaps using Morpheus (https://software.broadinstitute.org/morpheus/).

### 4.12. ALDH Activity

To assess ALDH enzymatic activity, the ALDEFLUOR kit (StemCell Technologies, 01700) was used. Cells were harvested and subjected to the ALDEFLUOR assay according to manufacturer’s instructions. The fluorescent ALDH-bright cells (ALDH-High) were detected in the green fluorescence channel (520–540 nm) of the Cyan ADP (Beckman Coulter, Brea, CA, USA). The gates were established using negative control cells that were stained with the ALDEFLUOR reagent and treated with the ALDH inhibitor, DEAB, provided with the ALDEFLUOR kit. Cytometric data analysis was performed with the FlowJo V10 software (Tree Star).

### 4.13. Tumorsphere Formation

MC38 cells were cultured in 3D as described above. At day 7, representative images of the wells were obtained using the ECLIPSE TE300 (Nikon, Minato City, Tokyo, Japan) microscope and the number of tumorspheres (>50 μm) formed was counted and plotted.

### 4.14. Drug Resistance Analysis

Cells were seeded in triplicates in 96-well flat-bottom plates (Merck, 0812; 5000 cells per well) or in 96-well clear flat bottom ultra-low attachment plates (Merck, CLS7007; 2000 cells per well). DMSO (vehicle control, Sigma, Saint Louis, MI, USA, 276,855), 5-FU (Selleckchem, Houston, TX, USA, S1209) and oxaliplatin (Selleckchem, S1224) were added to the corresponding wells at the indicated concentrations at day 0 (2D culture) or at day 4 (3D culture). Cells were cultured in the presence of DMSO, 5-FU or oxaliplatin for 72 h in total. Cell viability was assessed using the CellTiter-Blue^®^ Cell Viability assay (Promega, G8080) according to the manufacturer’s instructions. Measurements were performed on the FLUOstar Galaxy (MTX Lab systems, Bradenton, FL, USA, excitation 560 nm and emission 590 nm). Fluorescence intensity was transformed to the % of viable cells by multiplying the value obtained from each concentration of the tested drug with 100 and dividing it to the fluorescent value of the corresponding DMSO concentration control.

### 4.15. Cancer Stem Cell (CSC)-Enrichment Assay

Cells were cultured in 3D in the presence or absence of 5-FU as described above. For the FUT9-High Mix, 1.3 × 10^3^ MC38-MOCK cells and 0.7 × 10^3^ MC38-FUT9 cells were seeded in triplicates. For the FUT9-Low Mix, 1.9 × 10^3^ MC38-MOCK cells and 0.1 × 10^3^ MC38-FUT9 cells were seeded in triplicates. As a control, single cultures of 2 × 10^3^ MC38-MOCK or 2 × 10^3^ MC38-FUT9 cells were seeded in triplicates. At day 7, tumorspheres were dissociated with an enzyme-free cell dissociation buffer (Gibco, 13,151,014), passed through a 70 µm cell strainer (Falcon, 352,350) and single cell suspensions were obtained. Both at day 0 and at day 7, cells were washed and stained for Lewis^x^ as described above. Cytometric data analysis was performed with the FlowJo V10 software (Tree Star). The % of Lewis^x+^ cells (indicative of MC38-FUT9 cells) was calculated for each condition and time point. Results from day 7 were compared to results from day 0.

### 4.16. Cell Cycle Analysis

Cells were subjected to 48 h starvation using serum-free medium, after which serum-containing medium (10% FBS) was added to the cells for 0 h, 24 h or 48 h, respectively. The cells were harvested, washed and fixed with 4% PFA (Electron Microscopy Sciences, 15,710) at 4 °C for 30 min. At RT, 1 × 10^6^ cells per condition were stained with 1 mL staining solution containing 0.1% (*v/v*) Triton X-100 (Sigma, X100) and 1 µg/mL DAPI (Thermo Fisher, D1306) diluted in PBS for 10 min. The cell-cycle analysis was performed by flow cytometry using a Cyan ADP (Beckman Coulter, USA) or LSRII (BD Biosciences, USA) flow cytometers. Cytometric data analysis in the linear mode was performed with the FlowJo V10 software (Tree Star).

### 4.17. Cell Proliferation Assay

Cells were seeded in 96-well plates at different absolute numbers (50, 100, 200, 300, 400 or 500 cells/well) and cultured for 3 days (72 h) in 2D or 7 days in 3D as described above. The metabolic activity of the cells (indicative of the cell proliferation rate) was analyzed using the CellTiter-Blue^®^ Cell Viability assay (Promega, G8080) according to the manufacturer’s instructions. Measurements were performed on the FLUOstar Galaxy (MTX Lab systems, USA, excitation 560 nm and emission 590 nm).

### 4.18. Public Databases

The MuBase Oncology Database (Crown Bioscience; https://www.crownbio.com/oncology/oncology-databases/mubase) was used to access gene expression data (RNA-seq) of MC38-WT cells under the code: MC38-P0-3-161026. Expression levels were represented as log_2_ (fragments per kilobase of transcript per million mapped reads—FPKM). Values were acquired from the database and then plotted with the help of the Prism software (GraphPad V10 Software). The R2 genomics analysis and visualization platform (Academic Medical Center, Amsterdam, the Netherlands; https://hgserver1.amc.nl/cgi-bin/r2/main.cgi) was exploited to address gene expression data (microarray) of human colon cancer cell lines available in the Cancer Cell Line Encyclopedia under the code: Cell line CCLE Cancer Cell Line Encyclopedia-Broad-MAS5.0-u133p2. Cell lines derived from the rectum were excluded from the analysis. Expression levels were represented as log_2_ (fragments per kilobase of transcript per million mapped reads—FPKM). Values were acquired from the database and then plotted with the help of the Prism software (GraphPad V10 Software). Single-cell RNA sequencing (sc-RNA seq) data from 11 CRC primary tumors and their matched normal samples [56] were reanalyzed using the R package and the Seurat toolkit (https://satijalab.org/seurat/) [91] under default parameters. Clustering of epithelial cells based on FUT9 expression (*n* > 0 classified as positive, *n* = 0 classified as negative and *n* = FUT9 transcript number) was followed by tSNE dimensionality reduction (https://lvdmaaten.github.io/tsne/). For each cluster, the mean gene expression levels were obtained and pheatmap (https://cran.r-project.org/web/packages/pheatmap/index.html) was used for the generation of the final heatmap. More information about the gene sets used can be found in Table S7. The corresponding R script is available at https://github.com/MolecularCellBiologyImmunology/fut9-cancer.

### 4.19. Statistical Analysis

For the differential expression analysis, significance was assessed using the Benjamini–Hochberg false discovery rate (FDR) <0.05. Unless otherwise indicated, other statistical analyses were performed with the Prism software (GraphPad V10 Software). Statistical significance was determined by an unpaired Student’s *t* test for comparison of two groups, by a one way ANOVA for comparison of multiple groups and by multiple Student’s *t* tests for comparison of two groups across a range of drug dosages: * *p* < 0.05, ** *p* < 0.01 and *** *p* < 0.001.

## 5. Conclusions

In summary, our work suggests FUT9 as a potent reprogramming factor marking the acquisition of a stem-like and drug resistance phenotype by murine and human colon cancer cells. Given that FUT9 dictates the stemness in embryonic and neuronal stem cells too, its function seems to be evolutionary conserved and cell type-independent. Ultimately, this study warrants future research on the tumorigenic role of FUT9 in other cancer types and its potential exploitation as a CSC-specific biomarker for therapeutic targeting.

## Supplementary Materials

The following are available online at https://www.mdpi.com/2072-6694/12/9/2580/s1, Figure S1: Identification of transcription factor motifs (TMs) and transcription factors (TFs) relating neo-expression of FUT9/Lewis^x^ in MC38 cells to cancer stemness, Figure S2: MC38 cells acquire a stem cell-like phenotype upon FUT9/Lewis^x^ expression, Figure S3: MC38-glycovariants do not differ in their intrinsic cell proliferation capacity, Figure S4: FUT9 expression in human colon cancer cell lines is correlated with CSC-like features, Table S1: Differentially expressed genes in MC38 WT vs. MC38-MOCK and Differentially expressed genes in MC38-FUT9 vs. MC38-MOCK, Table S2: Motif enrichment analysis of upregulated genes in MC38-FUT9, Table S3: Target genes of the predicted transcription factors for the upregulated genes in MC38-FUT9, Table S4: Motif enrichment analysis of downregulated genes in MC38-FUT9, Table S5: Target genes of the predicted transcription factors for the downregulated genes in MC38-FUT9, Table S6: Gene ontogeny term enrichment analysis of differentially expressed genes in MC38-FUT9, Table S7: Genesets used for comparison between FUT9-Positive/Negative cells from healthy/tumor samples.
