# Supplementary material for: FUT9-Driven Programming of Colon Cancer Cells towards a Stem Cell-Like State
Source: Cancers (Basel). 2020 Sep 10;12(9):2580. doi: 10.3390/cancers12092580 (PMC7565653; doi:10.3390/cancers12092580)
Supplement: Supplementary file 1 [file cancers-12-02580-s001.zip › cancers-846103-supporting information-final/cancers-846103-supplementary-final.docx]

**
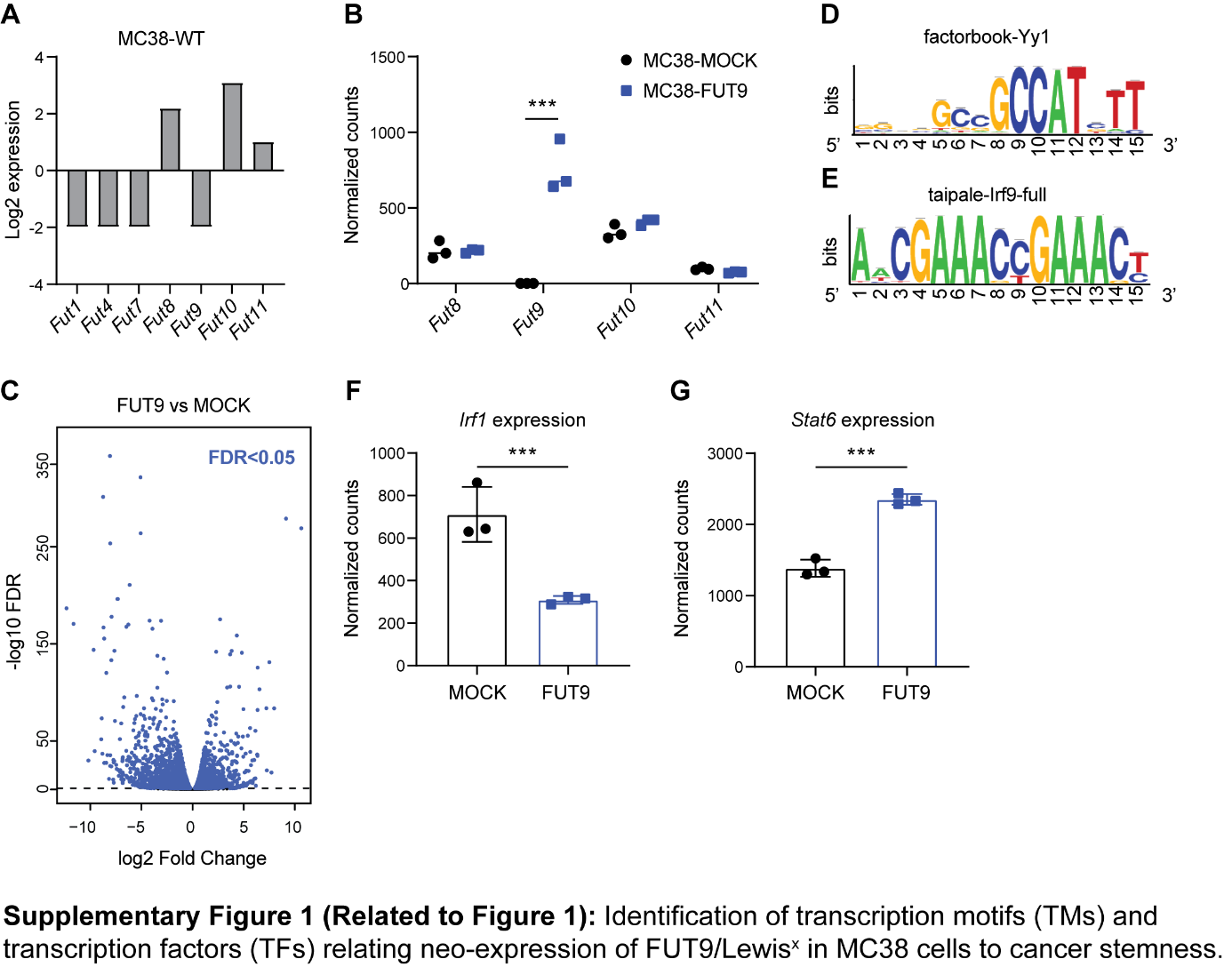
**

**Figure S1.** (Related to Figure 1). Identification of transcription factor motifs (TMs) and transcription factors (TFs) relating neo-expression of FUT9/Lewis^x^ in MC38 cells to cancer stemness (**A**) Log2 expression of fucosyltransferase (FUT) genes in MC38-WT cells according to the MuBase Oncology Database (Crown Bioscience). (**B**) Normalized counts for *Fut8*, *Fut9*, *Fut10* and *Fut11* in MC38-MOCK and MC38-FUT9 cells from the RNA-seq analysis. Statistical significance was assessed using Benjamini-Hochberg false discovery rate (FDR) <0.05. (*** *p* <0.001). (**C**) Differential expression analysis of the gene expression between MC38-FUT9 cells and MC38-MOCK cells. Depicted are the log_2_ fold change and FDR-adjusted p-value in a minus log 10 transformation. The dotted line was set at FDR = 0.05. (**D,E**) Enriched motifs found within the DEGs for the predicted transcription factors Yy1 (D) and Irf9 (E), respectively. The complete list of enriched motifs can be found in Supplementary Table S2 and Supplementary Table S4. (**F,G**) Normalized counts of the *Irf1* gene (F) and *Stat6* gene (G) in MC38-glyco-engineered cells. Statistical significance was assessed using Benjamini-Hochberg false discovery rate (FDR) <0.05. (****p* < 0.001).


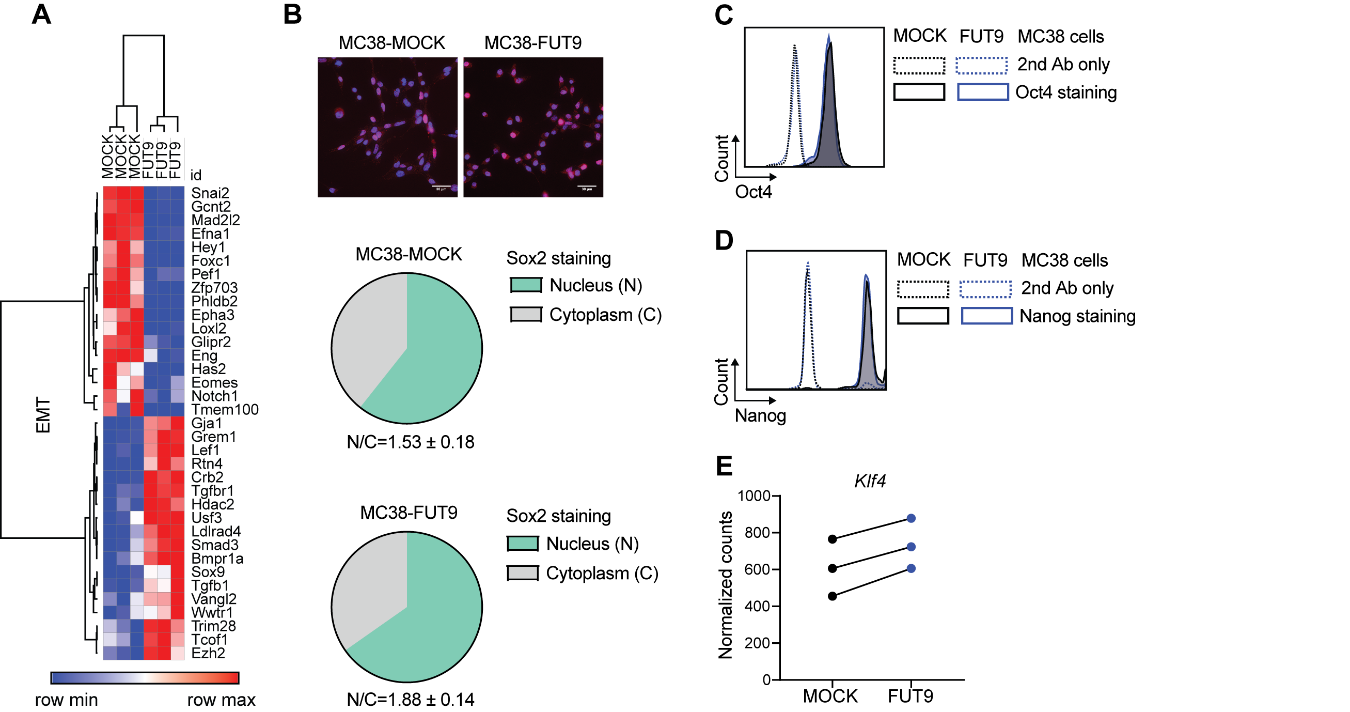


**Figure S2.** (Related to Figure 2). MC38 cells acquire a stem cell-like phenotype upon FUT9/Lewis^x^ expression (**A**) Expression of DEGs between MC38-MOCK and MC38-FUT9/Lewis^x^-expressing cells associated with epithelial to mesenchymal transition (EMT). (**B**) Intracellular staining of Sox2 in MC38-MOCK and MC38-FUT9 cells using immunofluorescence. Representative images are provided in the top panel. Sox2 staining is depicted in red color, while DAPI (staining of nuclei) is depicted in blue. Mean signal intensity of Sox2 staining and quantification of nuclear (green) to cytoplasmic (grey) ratio (± SD) for each cell line is shown in the bottom panel. Scale bar 30 μm. (C,D) Intracellular staining of Oct4 (C) and Nanog (D) in MC38 cells using flow cytometry (*n* = 1). (**D**) Normalized counts for the *Klf4* gene in MC38-glycovariants, data was extracted from the RNA-seq analysis.


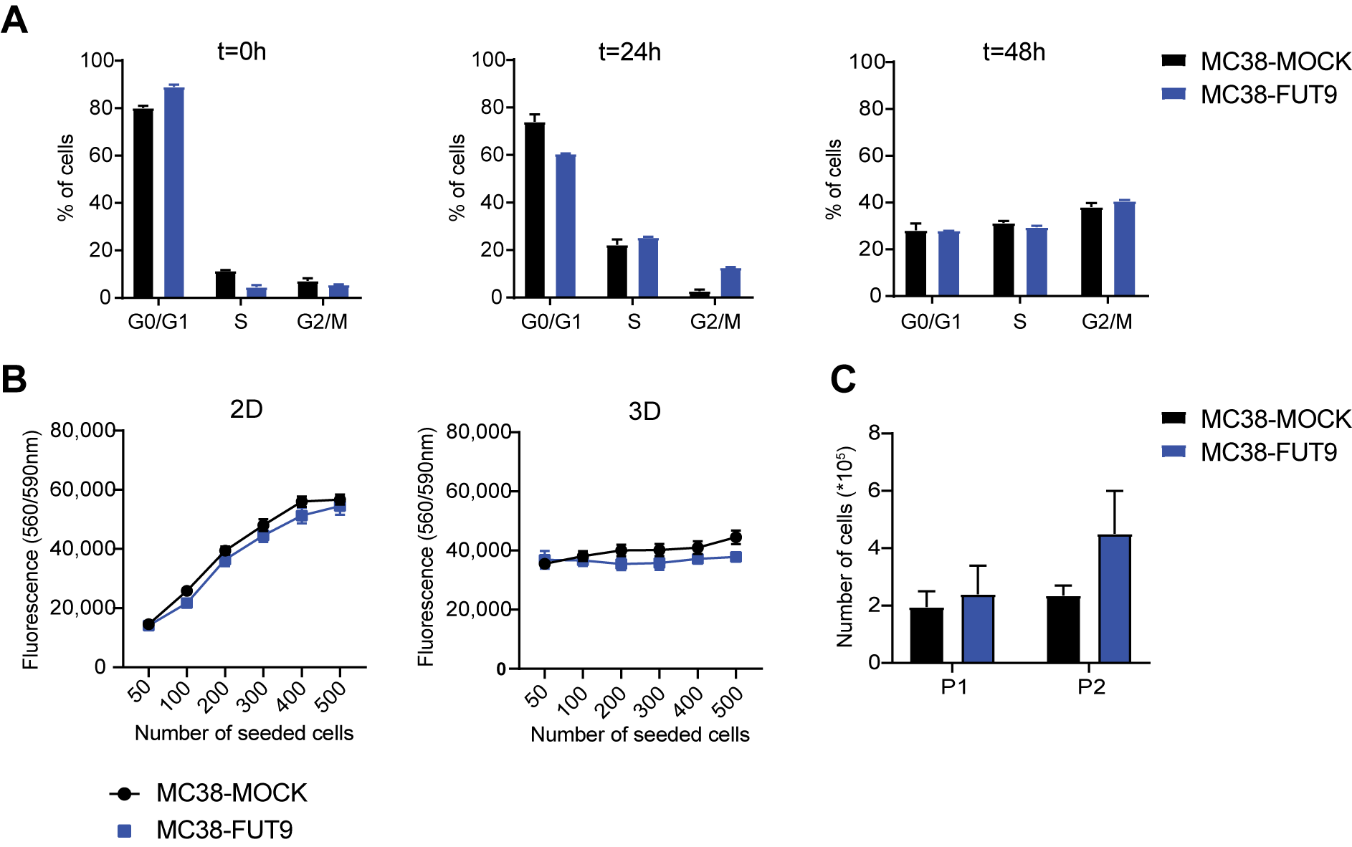


**Figure S3.** (Related to Figure 3). MC38-glycovariants do not differ in their intrinsic cell proliferation capacity (**A**) Cell cycle progression in MC38-glycovariants. Cells were subjected to serum starvation for 48 hours (synchronization) and then cultured in serum-containing (10%) medium for 0, 24, 48 hours. Afterwards, cells were harvested, fixed and stained with DAPI. The percentage of cells identified in the G0/G1, S and G2/M phase is depicted. Error bars represent SD. Data representative of two independent experiments. (**B**) Cell proliferation of MC38-MOCK and MC38-FUT9 cells cultured in 2D or 3D at different absolute numbers as assessed by the CTB fluorometric assay. Error bars represent SD; *n* = 3. (**C**) Number of MC38-MOCK and MC38-FUT9 cells obtained from dissociated tumorspheres after culture in 3D for two serial passages, passage 1 (P1) and passage 2 (P2). P1 corresponds to day 7, P2 corresponds to day 14. Data representative of two independent experiments.


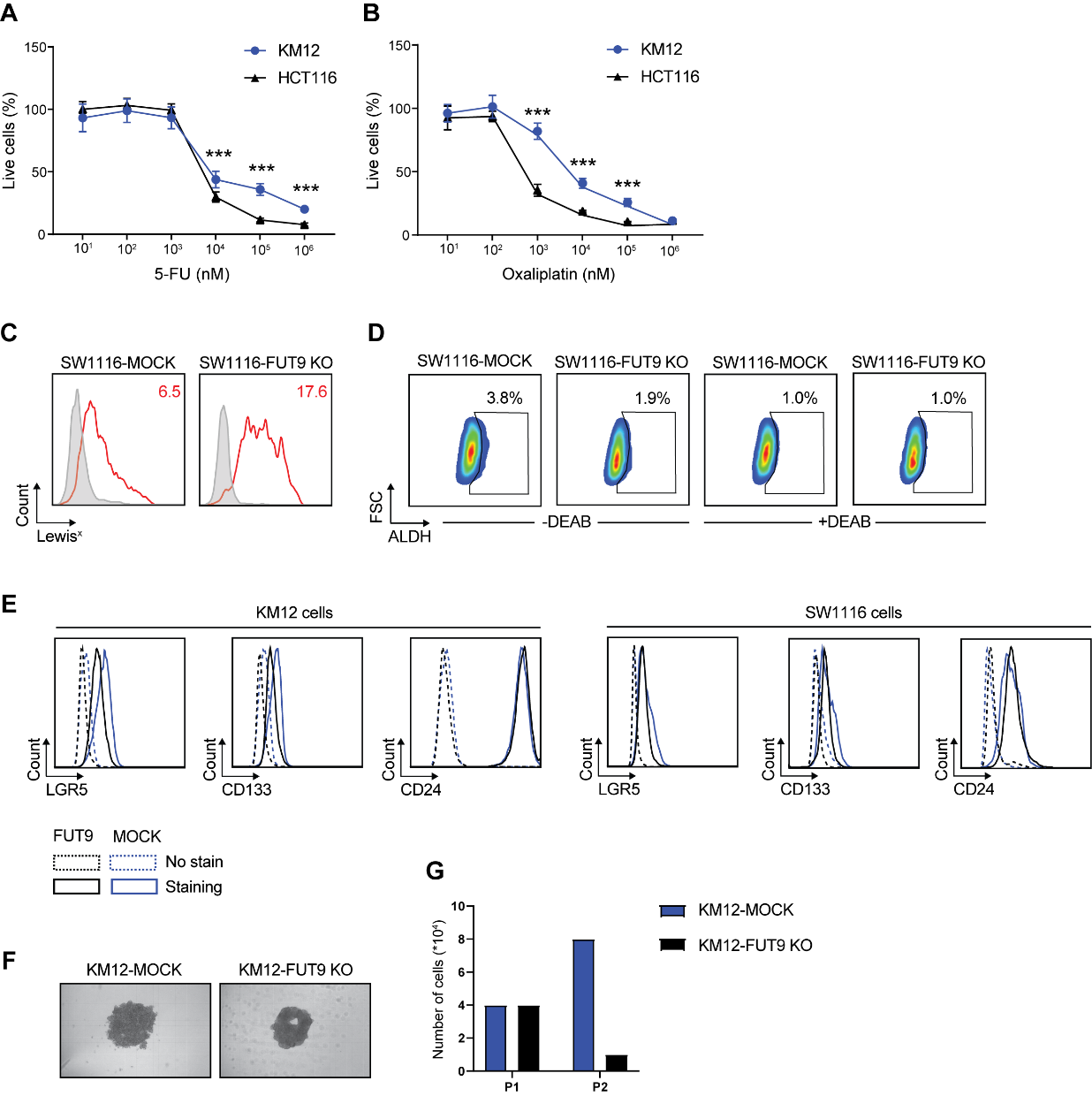


**Figure S4.** (Related to Figure 4): FUT9 expression in human colon cancer cell lines is correlated with CSC-like features (**A, B**) Viability of KM12 and HCT116 wild type cells upon treatment with indicated concentrations of 5-Fluoruracil (5-FU) (**A**) or Oxaliplatin (**B**) in 2D culture. Measurements were performed using the CTB fluorometric assay and values were normalized to the corresponding DMSO concentration for each cell line (set to 100%). Error bars represent SD; *n* = 3. Statistical significance was determined by multiple Student’s t tests (*** *p* <0.001). (**C**) Flow cytometric analysis of Lewis^x^ expression on the surface of SW1116 cells glyco-engineered with CRISPR-Cas9. Grey lines represent staining with the second antibody only, red lines represent Lewis^x^ staining. Numbers in red represent the mean fluorescent intensities (MFI) for Lewis^x^ staining normalized to the binding of the second antibody only. Histograms representative of two independent experiments are shown. (**D**) Representative dotplots of ALDH activity in SW1116-MOCK and SW1116-FUT9 KO cells measured by the ALDEFLUOR assay. The DEAB inhibitor of ALDH was used as a control for gating and the percentage of ALDH-High cells was calculated for each cell line. (E) Expression of LGR5, CD133 and CD24 in KM12 and SW1116 glycovariant cell lines as assessed by flow cytometry. Solid lines represent staining with the corresponding antibodies, while dotted lines represent the secondary antibody control. (**F**) Representative images of the KM12-glycovariant tumorspheres. (**G**) Number of KM12-MOCK and KM12-FUT9 KO cells obtained from dissociated tumorspheres after culture in 3D for two serial passages, passage 1 (P1) and passage 2 (P2). P1 corresponds to day 7, P2 corresponds to day 14.
