# Supplementary figures and images for: FUT9-Driven Programming of Colon Cancer Cells towards a Stem Cell-Like State
Source: Cancers (Basel). 2020 Sep 10;12(9):2580. doi: 10.3390/cancers12092580 (PMC7565653; doi:10.3390/cancers12092580)

# **IMMUNOBLOT IMAGES**

# FIGURE 1B

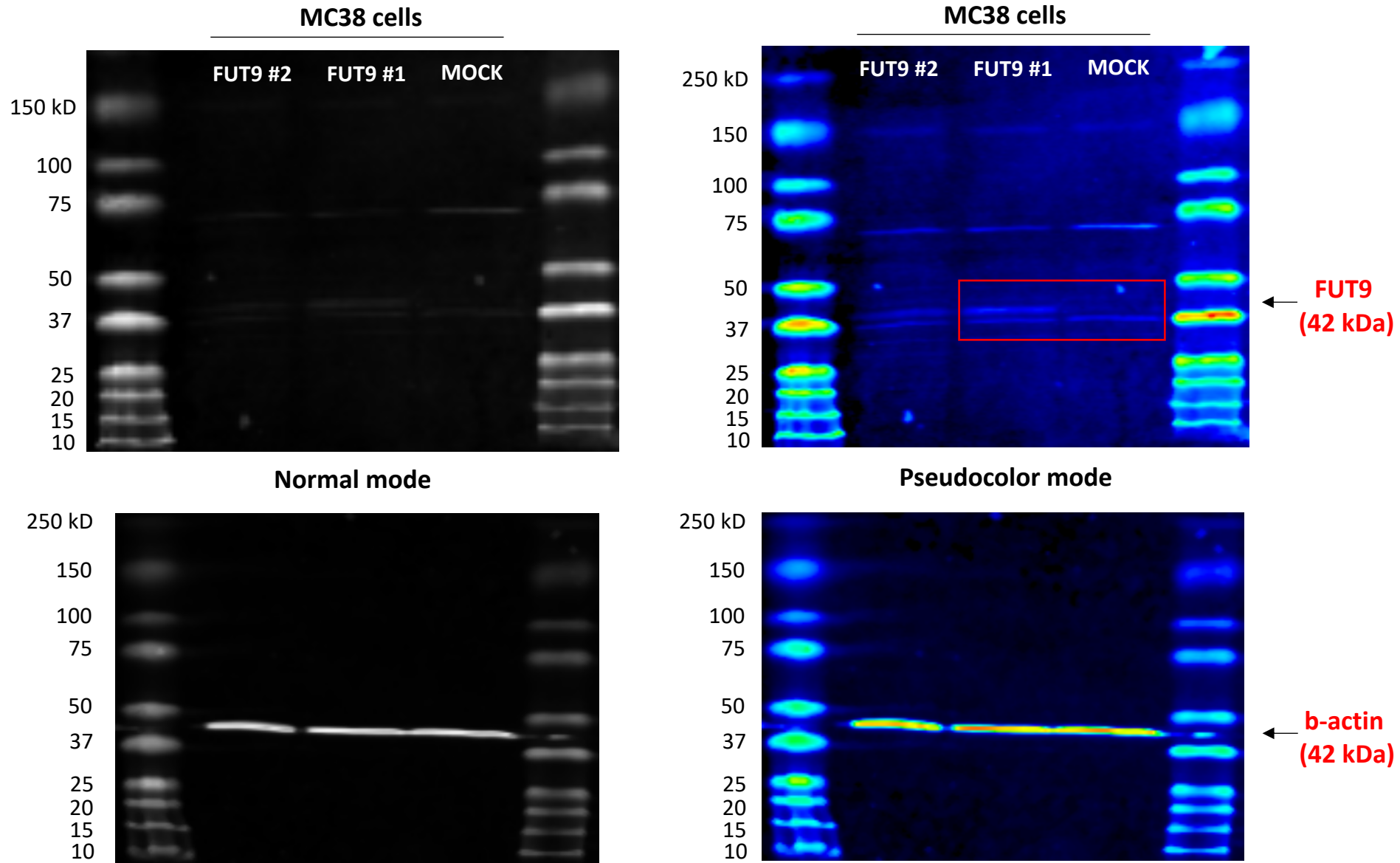

Supplement: Supplementary file 1 [file cancers-12-02580-s001.zip › cancers-846103-supporting information-final/cancers-846103-original_images.pdf]
